# Hotspots in *Plasmodium* and RBC Receptor-Ligand Interactions: Key Pieces for Inhibiting Malarial Parasite Invasion

**DOI:** 10.3390/ijms21134729

**Published:** 2020-07-02

**Authors:** Manuel Alfonso Patarroyo, Jessica Molina-Franky, Marcela Gómez, Gabriela Arévalo-Pinzón, Manuel Elkin Patarroyo

**Affiliations:** 1 Molecular Biology and Immunology Department, Fundación Instituto de Inmunología de Colombia (FIDIC), Carrera 50#26-20, Bogotá 111321, Colombia; jesmolina@uniboyaca.edu.co (J.M.-F.); mepatarr@gmail.com (M.E.P.); 2 School of Medicine and Health Sciences, Universidad del Rosario, Carrera 24#63C-69, Bogotá 112111, Colombia; gabriela.arevalo@urosario.edu.co; 3 Ph.D. Programme in Biomedical and Biological Sciences, Universidad del Rosario, Carrera 24#63C-69, Bogotá 112111, Colombia; aligomez@uniboyaca.edu.co; 4 Medicine Programme, Health Sciences Faculty, Universidad de Boyacá, Carrera 2ª Este#64-169, Tunja 150003, Colombia; 5 Biology and Microbiology Department, Sciences and Engineering Faculty, Universidad de Boyacá, Carrera 2ª Este#64-169, Tunja 150003, Colombia; 6 Receptor-Ligand Department, Fundación Instituto de Inmunología de Colombia (FIDIC), Carrera 50#26-20, Bogotá 111321, Colombia; 7 Universidad Nacional de Colombia, Carrera 45 # 26-85, Bogotá 111321, Colombia

**Keywords:** malaria, *Plasmodium*, receptor-ligand structure, structure activity relationship

## Abstract

Protein-protein interactions (IPP) play an essential role in practically all biological processes, including those related to microorganism invasion of their host cells. It has been found that a broad repertoire of receptor-ligand interactions takes place in the binding interphase with host cells in malaria, these being vital interactions for successful parasite invasion. Several trials have been conducted for elucidating the molecular interface of interactions between some *Plasmodium falciparum* and *Plasmodium vivax* antigens with receptors on erythrocytes and/or reticulocytes. Structural information concerning these complexes is available; however, deeper analysis is required for correlating structural, functional (binding, invasion, and inhibition), and polymorphism data for elucidating new interaction hotspots to which malaria control methods can be directed. This review describes and discusses recent structural and functional details regarding three relevant interactions during erythrocyte invasion: Duffy-binding protein 1 (DBP1)–Duffy antigen receptor for chemokines (DARC); reticulocyte-binding protein homolog 5 (*Pf*Rh5)-basigin, and erythrocyte binding antigen 175 (EBA175)-glycophorin A (GPA).

## 1. Introduction

Malaria is a disease caused by parasites from the phylum Apicomplexa and *Plasmodium* genus; they are characterized by two types of unique structures within the parasite: the apicoplast (a non-photosynthetic plastid) and the apical complex. The apical complex structure contains two specialized organelles called the rhoptries and the micronemes harboring high protein content needed for carrying out efficient host cell invasion [1].

**Figure 1:**
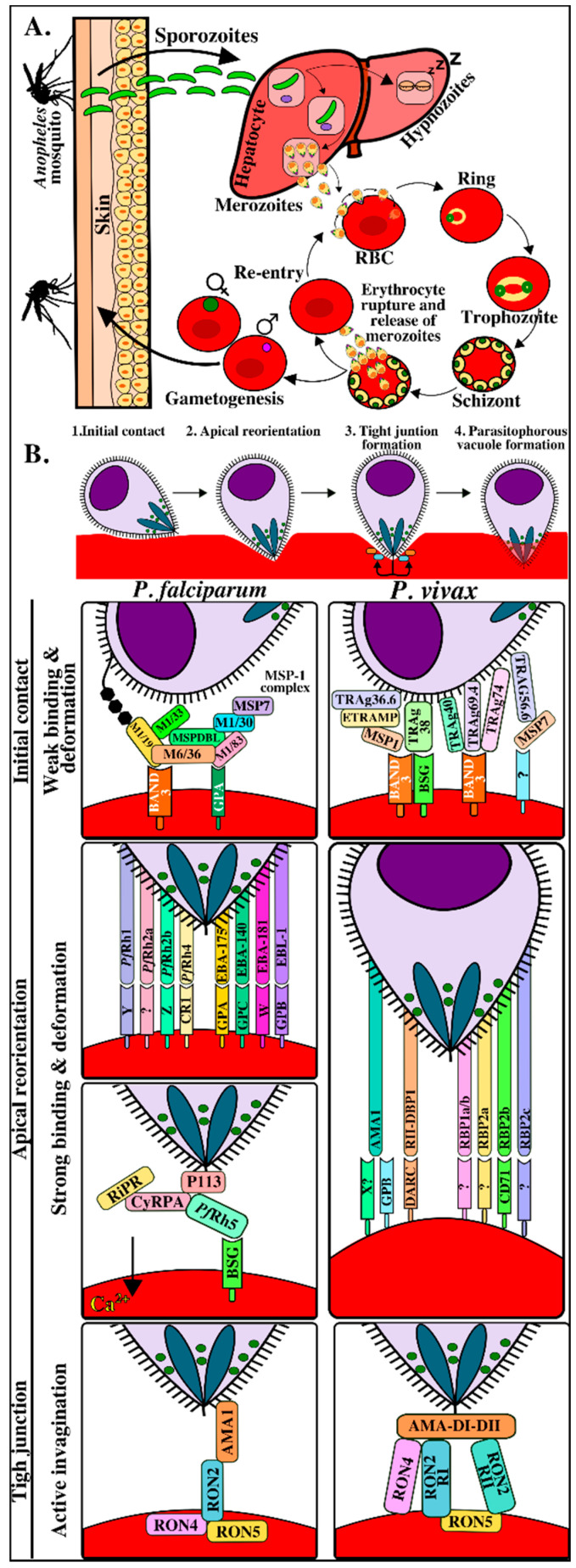
*Plasmodium* life cycle and erythrocyte invasion interactions. (**A**). The *Plasmodium* life-cycle has two phases in its hosts: the asexual phase in humans (vertebrates) and the sexual phase in *Anopheles* mosquitos (invertebrate). Female *Anopheles* mosquitos’ bites transmit the parasite in its sporozoite form into host dermis [19]. The inoculated sporozoites migrate through the bloodstream until reaching, invading, and developing within the hepatocytes. Some sporozoites may remain in a latent state within the hepatocytes (hypnozoites) during *Plasmodium vivax* invasion while others become transformed into a new parasite form called merozoite [34]. Once merozoites are released into circulation, they invade new red blood cells (RBC) and some become gametocytes which can be ingested by other mosquitos during a new bite. The parasite begins its sexual cycle in a mosquito to give rise to new sporozoites that will be transmitted to humans thereby starting the asexual cycle again in a vertebrate host. (**B**). Molecular events during *Plasmodium* merozoite invasion of RBC. Initial contact with target cells, merozoite apical pole reorientation to ensure direct contact with host cell membrane and establishing specific high-affinity interactions, tight junction formation acting as an anchor, and creating the parasitophorous vacuole, following parasite gliding motility towards target cells where they replicate, producing 30–50 new merozoites and subsequently following their cycle to invade other RBC [7,35]. M1/19, M1/30, M1/33, M1/83: merozoite surface protein 1–19 kDa, 30 kDa, 33 kDa and 83 kDa fragments, respectively; M6/36: merozoite surface protein 6–36 kDa fragment; MSPDBL: merozoite surface protein Duffy binding-like; MSP7: merozoite surface protein 7; ETRAMP: early transcribed membrane protein; TRAg36.6, TRAg38, TRAg40, TRAg69.4 and TRAg74: *Plasmodium* tryptophan-rich proteins 36.6, -38, -40, -69.4 or -74; BSG: basigin; ?: yet-unknown receptor; Rh1, Rh2a, Rh2b, Rh4 and Rh5: reticulocyte-binding protein homologues -1, -2, 2a, -2b, -4 and -5; EBA175: erythrocyte binding antigen 175; EBA140: erythrocyte binding antigen 140; EBA181: erythrocyte binding antigen 181; EBL: erythrocyte binding ligand; Y/Z/W: unidentified receptors; CR1: complement receptor 1, GPYA: glycophorin A; GYPB: glycophorin B; GYPC: glycophorin C; Ripr: *Pf*Rh5-interacting protein, CyRPA: cysteine-rich protective antigen; AMA1: apical membrane antigen-1, DARC: Duffy antigen receptor for chemokines, RII-DBP1: Duffy binding protein 1 - region II; RBP1a/b: reticulocyte-binding protein 1a/b; RBP2a: reticulocyte-binding protein 2a; RBP2c: reticulocyte-binding protein 2c; CD71: transferrin receptor 1; RON2: rhoptry neck protein 2; RON4: rhoptry neck protein 4; RON5: rhoptry neck protein 5; AMA1 DI-II: apical membrane antigen-1 domain I-II; RON2 RI: rhoptry neck protein 2 - region I; RON2 RII: rhoptry neck protein 2 - region II.

The five human malaria parasites’ life-cycle is extremely complex and involves two asexual life stages within a human host and a sexual stage within a female *Anopheles* mosquito. The parasite undergoes a series of synchronized morphological, transcriptional, and protein expression changes (i.e., different proteins are expressed depending on the parasite form), thereby enabling efficient invasion (of two different target cells) and escaping the human immune system [2,3]. Once the first parasite form (called a sporozoite) enters a human host, it reaches the hepatic cells by gliding motility and by cell traversal mechanisms where it uses different proteins to bind receptors on human hepatic cells, thus gaining access to them. The sporozoites divide and become differentiated there to produce hundreds of merozoites, thereby starting the invasion of human erythrocytes (blood or intra-erythrocyte phase) (Figure 1A). The parasites develop repeated cycles of replication, exit, and re-invasion of new erythrocytes during this phase, involving the parasite passing from ring to trophozoite and then to schizont form (Figure 1A). This intra-erythrocyte stage takes 48–72 hours, depending on the *Plasmodium* species, and gives rise to the disease’s clinical manifestations [4,5]. A small percentage of parasites transform into gametocytes during this stage and are taken up during a blood meal by another feeding *Anopheles* mosquito (Figure 1).

In contrast to viral and intracellular bacteria invasion, *Plasmodium* actively invades erythrocytes without having to depend on host cells’ capture routes [6]. Information regarding this process has been obtained from studies on *Plasmodium knowlesi* and *Plasmodium falciparum* and has been conceptually divided into four phases (Figure 1B) [7,8,9,10,11,12,13,14]. The first involves initial contact through weak and transitory interactions associated with waves of erythrocyte membrane deformation mediated by merozoite surface proteins’ (MSPs) interaction with erythrocyte surface [15,16]. Erythrocytes become more deformed after binding and, in the second phase, the parasite reorients its apical pole to come into direct contact with the host cell membrane. This is called reorientation and, in *P. falciparum*, is mediated by the actin-myosin motor and by the early release of erythrocyte-binding antigens (EBAs) from the micronemes and reticulocyte-binding protein homologs (Rhs) from the rhoptries, binding with high affinity to a wide range of host cell receptors [17,18]. Such reorientation results in the formation of a strong pre-tight junction in which the reticulocyte-binding protein homolog 5 (Rh5) protein (only found in *P. falciparum* and *P. reichenowi*) becomes translocated to erythrocyte membrane, forming a protein complex with other parasite antigens, to bind to the basigin receptor (CD147) on erythrocytes [19], thereby triggering the release of rhoptry neck (RON) proteins [16]. Such release marks the start of the third phase enabling RON transfer within erythrocyte membranes to initiate tight junction (TJ) formation characterized by a ring-like structure which starts from the parasite’s apical pole and moves progressively towards its posterior end as it enters host cells inside the parasitophorous vacuole (PV) [20]. It has been found that this is mediated by RON protein translocation towards host cell cytosol in *Toxoplasma gondii* and *P. falciparum*. RON2 becomes inserted into the cell membrane to act as a receptor for apical membrane antigen 1 (AMA1) protein and this interaction provides a strong anchoring point, enabling successful parasite invasion [21,22]. The fourth phase involves parasite access to host cells where it becomes housed within a PV to begin infection and replication (Figure 1B and Table S1).

As well as the wide repertoire of receptor-ligand interactions taking place, it is worth highlighting macromolecular complex formation between parasite proteins in *P. falciparum* and in *Plasmodium vivax* (supported by less evidence in the latter) which could promote interaction with receptors on merozoites (Figure 1B) [23,24,25]. In *P. falciparum*, the MSP-1-mediated interaction platform has been one of the most widely characterized complexes; it is capable of interacting with MSP6 and MSP7 proteins, the high molecular weight rhoptry protein 3 (RhopH3), rhoptry associated protein 1 (RAP1), RAP2, MSP1DBL1, MSP1DBL2 and a co-ligand complex with MSP9 [23,24]. MSP1 fragments interact with glycophorin A, band 3, and yet-to-be-identified erythrocyte membrane-associated receptors [26,27]. In addition, *Pf*Rh5 forms a complex with protective cysteine-rich antigen (CyRPA) and Rh5-interacting protein anchored to parasite surface by the P113 protein via a glycosylphosphatidylinositol (GPI) lipid anchor to interact with the basigin receptor. The TJ is a macromolecular complex consisting of rhoptry neck proteins (RON2-RON4-RON5) and AMA-1 (Figure 1B).

Understanding the molecular mechanisms involved in merozoite binding to and invasion of erythrocytes has been one of the main approaches in basic malaria research aimed at developing drugs and vaccines against this parasitosis [7,8]. Spectroscopic techniques such as nuclear magnetic resonance (NMR), nuclear X-ray crystallography, hydrogen-deuterium exchange mass spectrometry (HDX-MS) and mutational mapping linked to physical techniques such as surface plasmon resonance (SPR) and isothermal titration calorimetry (ITC) have provided detailed information regarding the molecular structure and interaction regions of some key proteins involved in erythrocyte invasion [28,29,30,31,32]. Critical interaction regions between parasite ligands and host cell receptors have been defined from the structures of two of the most important *Plasmodium* species worldwide: *P. falciparum* and *P. vivax*. This has also enabled identifying functional and non-functional epitopes from ligand-neutralizing and/or inhibitory antibody (Ab) complexes [29,30,32,33]. Combining both approaches (receptor-ligand and antigen-antibody) highlights small ligand regions which are critical for parasite invasion, i.e., hotspots; these can be used for the rational design of future vaccines components or treatment against malaria infection.

## 2. *Plasmodium vivax* and Its Main Receptor-Ligand Interaction

*P. vivax* is the second most important malaria-causing species worldwide after *P. falciparum* [36]. This parasite species has tropism for stage I-III reticulocytes expressing two surface receptors during the blood stage: Duffy antigen receptor for chemokines (DARC) and transferrin receptor 1 (TfR-1 or CD71) [37,38]. DARC was the first receptor identified for *P. vivax* invasion via its specific interaction with Duffy-binding protein 1 (DBP1) [39]. Although there are discrepancies regarding whether DARC-DBP1 interaction is completely required for invasion (mainly due to reports showing that Duffy-negative individuals have been diagnosed with *P. vivax* infection throughout Africa) [40], such interaction has undoubtedly been the most and best studied in *P. vivax*.

**Figure 2:**
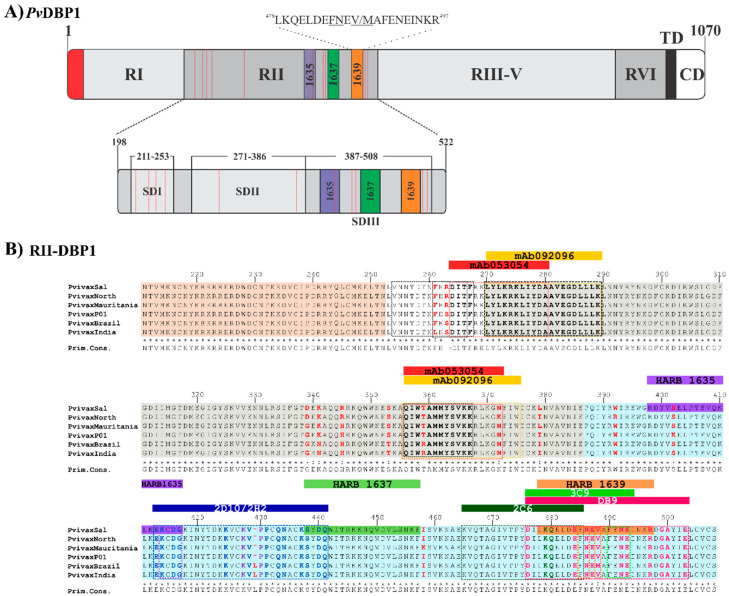
DBP1 representation. (**A**) Schematic representation of DBP1 primary structure showing each region’s length and representing the three HARBPs located in SD3. HARBP 1639 aa sequence is shown. The highlighted residues are critical binding residues. The red dotted lines indicate the location of the Cys involved in disulfide bridges. RI (region I), RII (region 2), RIII-V (region 3 to region 5), RVI (region 6), TM (transmembrane domain), CD (cytoplasmic domain) [42,43]. (**B**) Alignment of RII-DBP1 from the main *P. vivax* strains having different geographical locations. RII-DBP1 subdomains: S1 ^211^N-L^253^ (pink), S2 ^271^Y-E^386^ (gray), ^387^S3-P-S^508^ (cyan). DBP1 residues involved in binding to DARC: RII-DBP1 ^261^F – T^266^, ^270^L – K^289^, and ^356^Q – K^367^ (The primary DARC binding interface) and ^254^V-F^267^ (secondary DARC binding interface) (black box) highlighting the critical contact residues [29,44]. Amino acid composition variations are highlighted in red. mAb 053054: ^264^D-A^281^, ^356^Q-N^372^ (red box) and 092,096 epitopes: ^249^E, ^270^L-K^289^ and ^356^Q-W^375^ (yellow box) overlapping in SD2 [28]. mAb 2D10 and 2H2 epitopes (blue box), highlighting critical contact residues (2D10-blue and 2H2-dark purple) [50]. mAb 2C6 epitope: ^265^K – F^486^ (dark green box), highlighting critical contact residues. mAb DB9 epitope: ^476^D – E^503^ (magenta box), highlighting critical contact residues [33]. mAb 3C9 epitope: ^476^D – E^493^ (green box), highlighting critical contact residues [55] HARBPs 1635: ^398^R – G^417^ (purple), 1637: ^438^S – F^457^ (light green) and 1639: ^478^L –R^497^ (apricot) located in neutralizing epitopes’ SD3 region [56]. HARB 1639 critical binding residues matching mAb 3C9 are highlighted (arrows). HARBPs: high activity reticulocyte binding peptides. mAbs: monoclonal antibodies. Cys: cysteine.

DBP1 is a protein member of the erythrocyte binding-like (EBL) superfamily found in species such as *P. falciparum* and *P. knowlesi* [41]. The members of this family contain one or two Duffy binding-like (DBL) cysteine-rich (region II) extracellular domains, a second extracellular cysteine-rich domain (region VI), a transmembrane domain and a short cytoplasmatic domain (Figure 2A). Initial studies have led to conclude that the amino acid (aa) sequence from residue 198 to 522 (called RII) is responsible for the specific interaction with DARC [42,43]. RII-DBP1 becomes gradually coupled to DARC to create a stable heterotetramer consisting of two DBP molecules and two DARC molecules [29,44].

It is clear that RII-DBP1 can induce a natural human immune response (meaning that DBP1 is recognized by a host’s immune system during natural infection) and that the heterotetramer’s interaction interface is a neutralizing Ab response target [28,29,45]. This complex has high polymorphism (one of the main immune response evasion mechanisms), several having been found in RII-DBP1. Analyzing this region’s genetic diversity in *P. vivax* field isolates from geographically remote areas, such as Papua New Guinea, South Korea, Colombia, Brazil, and Thailand, has confirmed high RII-DBP1 polymorphism, but highly conserved cysteine residues [46,47,48]. Such analysis has revealed that RII-DBP1 has around 127 polymorphic sites, resulting in 193 haplotypes [49] having a ten-fold higher substitution rate than the rest of the molecule (i.e., such regions tend to fix more nucleotide changes than other regions) [50,51]. Interestingly, aa substitutions at D^384^G, K^386^(N/Q), N^417^K, L^424^I, W^437^R, and I^503^K were found in Papua New Guinean and Colombian isolates, showing that many similar alleles are widely distributed among *P. vivax* from different geographical areas [46,52]. Although it has been suggested that polymorphisms in RII-DBP1 do not significantly alter host-parasite binding, some of them alter immune recognition of DBP1 [53]. Consequently, the polymorphic nature of DBP1, particularly RII-DBP1, represents a serious problem negating this antigen’s use as an essential molecule in a vaccine against *P. vivax*. Functional analysis of reticulocyte binding, together with identifying epitopes eliciting broadly neutralizing Abs (bnAbs), correlated at a structural level, has exposed some other regions (analyzed below in this review) which could become alternate hotspots contributing towards the rational design of DBP-based vaccines and therapies. This would overcome the inconvenience of having a ten-fold higher substitution rate, bearing in mind that DBP1 is the most clinically representative and advanced *P. vivax* blood-stage antigen.

**Figure 3:**
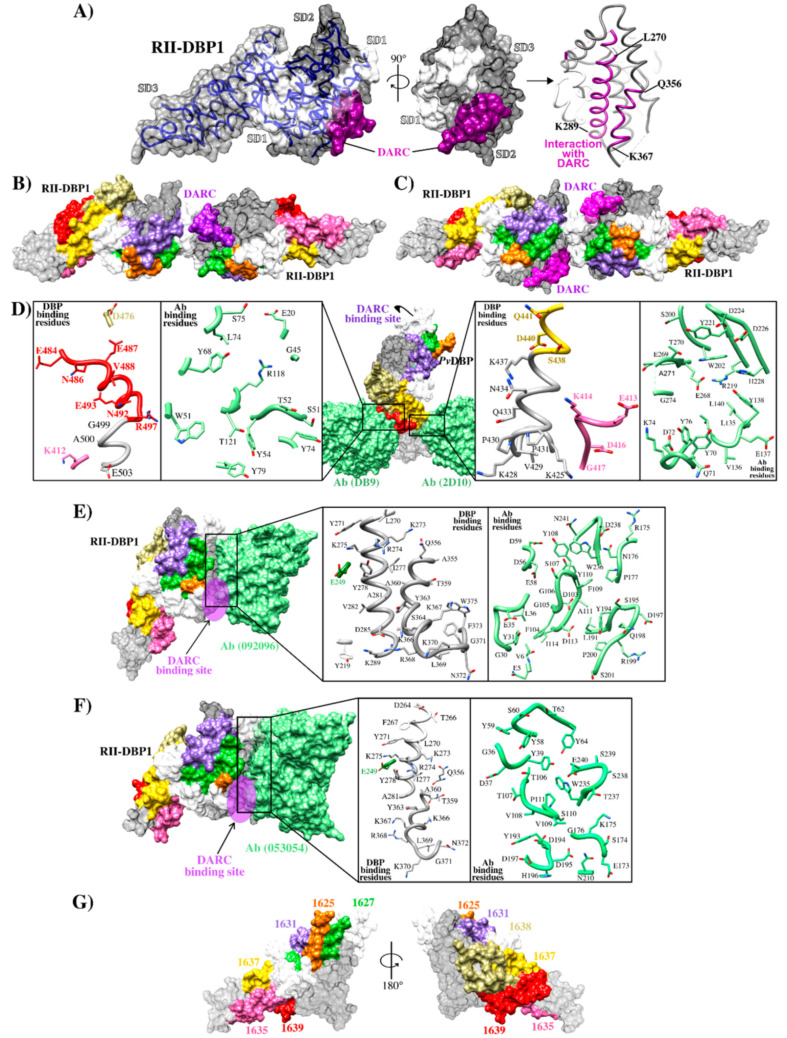
RII-DBP1 structure and its interaction with DARC (**A**) RII-DBP1, showing its three subdomains (SD1, SD2 and SD3) and the DARC binding region [42,43]; (**B**) RII-DBP1 two-ligand heterotrimer structure and a monomer from the DARC receptor; (**C**) RII-DBP1:DARC heterotetramer structure reconstructed from PDB 3RRC, 4NUU, 4NUV, 5F3J, 6OAN, 6OAO and 6R2S [28,29,33,44,57];.(**D**) RII-DBP1 contact points with mAb DB9-2D10, zooming in on interacting residues highlighting HARBPs: 1635 (pink), 1637 (yellow), and 1639 (red) (PDB 6R2S and 5F3J) [33,57]; (**E**) DBP1/DARC points of contact with mAb 053054, zooming in on interacting residues (PBD 6OAN) [28]; (**F**) DBP1-DARC contact points with mAb 092096, zooming in on interacting residues (PDB6OAO) [28]; (**G**) RII-DBP1 including HARBPs 1625 (orange), 1627 (green), 1631 (blue), 1635 (pink), 1637 (yellow) and 1639 (red) [56].

RII-DBP1 is an elongated boomerang-shaped molecule having an antiparallel β-hairpin turn close to the N-terminus. This region consists of three subdomains stabilized by intra-subdomain disulfide bridges. Subdomain 1 (SD1) consists of residues ^211^N–L^253^ and has two intra-subdomain disulfide bridges: ^217^C–C^246^ and ^230^C–C^237^. Subdomain 2 (SD2) consists of residues ^271^Y–E^386^ and has an intra-subdomain disulfide bridge (^300^C–C^377^) while subdomain 3 (SD3) consists of residues ^387^P–S^508^ and has three disulfide bridges: ^415^C–C^432^, ^427^C–C^507^ and ^436^C–C^505^ [29] (Figure 2A and Figure 3). NMR, X-ray crystallography and ITC have determined that the RII-DBP1 region for binding to DARC consists of residues 256-426 covering the whole of SD2 and has all the dimerization components required for engaging a putative sulfotyrosine (^41^Tyr) on DARC, ^273^K and ^356^Q being critical residues in the DARC-binding pocket [44,54]. ^270^L-K^289^, ^356^Q-K^367^, and ^261^F-T^266^ have been defined as critical RII-DBP1 residues directly contacting the DARC receptor (Figure 2B and Figure 3A–C) [41]. Such information provides this interaction’s molecular components and mechanistic role, thus expanding biological knowledge regarding receptor-ligand interactions in *P. vivax*.

Biophysical studies have shown that a non-sulfated DARC construct functionally binds to and is capable of inducing RII-DBP1 dimerization [44], suggesting that regions outside of the sulfotyrosine residues play an important role in binding interaction. Interestingly, it has been identified that DBP1 SD2 is characterized by having mainly polymorphic residues grouped around DARC dimerization and binding interphase in modeled RII-DBP1 structure [58]. It has been observed that nucleotide diversity within RII-DBP1 was universally highest in a region matching a previously identified inhibitory epitope (termed the DEK epitope) when examining nucleotide diversity patterns regarding individual geographical locations [50]. SD3, located at the opposite end of SD1-SD2, had very low nucleotide diversity in all populations [58]. Attention must thus be paid to regions having low substitution rates which could reduce strain specificity. Researchers have thus focused their efforts on producing Abs blocking receptor-ligand interactions or elucidating the structural bases defining the neutralization mechanisms of naturally acquired Abs or those induced by vaccination [50,57].

Ntumngia et al., produced monoclonal Abs (mAb) targeting the RII-DBP1 region capable of blocking RII-DBP1 binding to human erythrocytes [50]. Such Abs called 3C9, 2D10, 2H2 and 2C6 targeted a region located in SD3 (Figure 2B and Figure 3D) [50,55]. Ab 2D10 specifically recognized a conformational epitope between residues 413–417 and 425–441. Studies involving small-angle X-ray scattering (SAXS), HDX-MS, and ELISA ascertained that Ab 2H2 shared overlapping binding regions on DBP1 with Ab 2D10 (Figure 2B and Figure 3D). Ab 2C6 recognized an epitope located between residues 465–485. Interestingly, the epitopes recognized by these mAbs occurred in a highly conserved region [57]. Although these Abs were not directly targeting DBP1-DARC interaction interface, it has been suggested that their mechanism of action would be steric hindrance preventing RII-DBP1 approaching erythrocyte surface [33].

Studies orientated towards mapping specific DBP1 reticulocyte binding regions have found that seven conserved and semi-conserved peptides obtained by solid-phase peptide synthesis and identified by serial numbering (called high activity reticulocyte binding peptides - HARBPs) specifically interacted with reticulocyte surface [56]. Interestingly, several of them were located in regions different to those involved in DBP1 dimerization and DARC binding, suggesting that other DBP1 regions mediate interaction with reticulocytes and that they could be considered when designing vaccines against species. Three HARBPs, 1635 (semi-conserved) (aa 398–417), 1637 (conserved) (aa 438–457), and 1639 (semi-conserved) (aa 478–497) (Figure 3G), formed part of the regions recognizing binding-neutralizing Abs 2D10, 3C9, 2H2, and 2C6. HARBPs 1635 and 1639 inhibited recombinant protein RII-DBP1 binding to human reticulocytes, showing that the Abs’ inhibition mechanism could be due more to receptor-ligand blocking than steric hindrance. Replacing each aa in HARBP 1639 by glycine showed that residues ^485^F, ^486^N (conserved amongst strains), and ^488^V were critical to reticulocyte binding and coincided with critical Ab 3C9 contact residues and partially with Ab 2C6 contact region (Figure 2B and Figure 3D). Studies regarding enzyme treatment of human erythrocytes and peptide-receptor cross-linking have ascertained have HARBP 1639 interacted with a chymotrypsin treatment-resistant receptor of around 40 kDa that did not coincide with the DARC binding profile (sensitive to chymotrypsin treatment) [56].

Recent studies have emphasized the theory that *P. vivax* could have alternative invasion routes due to their ability to invade DARC-negative erythrocytes [59,60]. However, it has still not been elucidated whether using alternative routes could be mediated by other parasite proteins or DBP1. Genomic analysis has reported that DBP1 duplication has occurred more frequently in patients from Madagascar (DARC-negative) compared to other regions, suggesting increased DBP1 production as a compensatory mechanism for binding to other receptors on the membrane [61]. Although such statements are controversial, analyzing DBP1 erythrocyte binding regions that did not coincide with DARC binding regions and Abs blocking such binding have revealed other DBP1 regions involved in adhesion.

Reviewing *P. vivax* vaccine candidate development has shown that RII-DBP1-based vaccines have progressed through Phase I clinical trials (Table 1). Abs against RII-DBP1 were obtained in some cases which were maintained for more than 100 days following the three immunization doses and had pronounced DBP1-DARC binding inhibiting capability [62,63]. It has been reported that vaccination with this antigen has induced a strain-transcending Ab response and blocked receptor binding by different DBP1 allele variants [62]. DBP1 antigenicity studies have shown that high, naturally-acquired Ab titers can block RII-DBP1-DARC interaction in vitro and are associated with a reduced risk *P. vivax* infection [64,65]. Although Ab induced by a natural immune response and vaccination might differ, detailed analysis of epitopes recognized by Abs induced by both mechanisms which can block DBP1-DARC interaction (sometimes with different DBP1 allele variants) could reveal hotspots on which future intervention points should be focused. This information, combined with receptor-ligand interaction studies, represents an essential complement for highlighting interesting regions in *P. vivax* ligands.

In line with this idea, human-derived neutralizing mAbs produced by sorting individual RII-DBP1 specific B-cells from a Cambodian patient with naturally acquired immunity has revealed that mAbs 053.054 and 092.096 could prevent binding between RII-DBP1 and the N-terminus of DARC receptor and neutralize *P. vivax* invasion ex vivo [28]. Structural and HDX epitope-mapping data has demonstrated that the 053.054 epitope spans residues ^249^E, ^264^D, ^281^A, and ^356^Q, ^372^N of helices in SD2 [28]. These are key residues in the DARC binding interface where ^264^D is directly in contact with DARC and ^356^Q forms part of the DARC sulfotyrosine binding pocket [29,44]. The epitope recognized by mAb 053.054 overlapped the epitope for 092.096, consisting of ^219^Y, ^249^E, ^270^L–K^289^, and ^356^Q–W^375^ (Figure 3E,F).

Cloning studies of a panel of Abs from volunteers immunized with RII-DBP1 revealed markedly strain-dependent differences in anti-RII-DBP1 mAb potency. Only one of the ten mAbs evaluated (DB9) inhibited invasion by 65–90% in ten of the eleven *P. vivax* isolates [33]. This Ab induced powerful inhibition of *P. knowlesi* transgenic growth in inhibition assays and inhibited the binding of all five RII-DBP1 variant alleles to DARC. The DB9 epitope was contained in SD3 (Figure 2B), which is one of the domain’s most conserved regions; however, this sequence was distant from the DBP1-DARC contact and dimerization site and recognized residues ^477^D, ^485^E, ^487^N, ^488^E, ^489^V, ^493^N, ^494^E, ^498^R, ^499^G, ^500^A, and ^504^E (Figure 3D). It overlapped the whole HARPB 1639 sequence and the epitopes recognized by mAb 3C9 and 2C6 [50,56]. This hotspot (located in SD3 between residues 476–500) is a promising target considering that it contains regions specifically interacting with reticulocytes, mAbs targeting it can highly inhibit parasite entry to and interaction with reticulocytes, and has fewer polymorphic changes than SD2 [28,33,56,58]. It has been reported that Abs targeting epitopes in this subdomain can inhibit erythrocyte binding by different RII-DBP1 allele variants [55].

## 3. *Plasmodium falciparum*: A Thousand and One Invasion Routes

*P. falciparum* is the most important malaria-producing species worldwide and has been the most widely studied of the five species infecting humans [71]. Transcriptional studies have shown that around 50–60 genes encoding proteins that might mediate erythrocyte invasion are over-expressed in schizonts compared to other parasite forms, such as rings and trophozoites [3]. Research groups have thus focused their efforts on identifying, characterizing, and functionally evaluating *P. falciparum* antigens located on this species’ membrane and apical organelles and their participation in establishing interactions with receptors on erythrocyte membrane [71,72,73]. It has thus been established that initial interactions between the parasite and erythrocytes are mediated by the MSP-1 protein complex [7,23]. Such interaction leads to strong erythrocyte deformation and merozoite reorientation mediated by different invasion routes in which EBA and Rh family ligands participate. Depending on receptor availability, *P. falciparum* will express a combination of EBAs or Rhs for interacting with target cells; the interaction between EBA-175-glycophorin A (GPA) is however the preferential *P. falciparum* invasion route (Figure 1) [19,74,75]. This is followed by ternary complex formation between *Pf*Rh5 and CyRPA and Ripr interacting with the BSG receptor on erythrocytes, thereby initiating the rhoptry release stage and pre-TJ formation facilitating RON2 complex (RON2, 4 and 5) insertion into erythrocyte surface [25,76,77]. The RON2 complex thus acts as AMA1 receptor giving rise to the TJ formation which slides as the parasite accesses host cells. Structural evidence is crucial for finding the key intervention points in such complex and coordinated invasions.

Previous studies have shown that the structural elucidation of internal *P. falciparum* therapeutic targets could also open up another focus for exploration. For example, studies combining the search for *P. falciparum* 20S proteasome-specific inhibitors with high-resolution structural determination of the 20S proteasome by cryo-EM has provided valuable information that can be used to assist in designing improved proteasome inhibitors to be developed as next-generation antimalarial drugs [78]. Taking such interesting results into account regarding the advance of new antimalarial drugs, adopting the same strategy with ligands and receptors involved in the different steps of erythrocyte invasion could highlight new regions of vaccine interest. Although each step is essential, elucidating the structural relationships/interactions between *Pf*Rh5-BSG and EBA-175-GPA has aroused great functional interest.

### 3.1. The Essential Interaction: PfRh5-Basigin

*P. falciparum* reticulocyte-binding protein homolog 5 (*Pf*Rh5) is a prominent blood-stage antigen which is solely expressed in the subgenus *Laverania* and is an exceptional member of the Rh family as it is the only *Pf*Rh gene where attempts at knock-out have not been successful [79]. It has a low molecular weight, lacks transmembrane domains and Abs targeting this protein can neutralize a wide range of laboratory strains adapted in culture and *P. falciparum* clinical isolates [79]. Such a characteristic, added to limited genetic polymorphism concerning multiple *P. falciparum* laboratory strains and geographic isolates regarding other candidates has led to a significant amount of research on this protein in the field of antimalarial vaccines [80,81,82]. Although few studies have involved using clinical isolates, previous *Pfrh5* genetic diversity studies using isolates from Nigeria and Mali have coincided by confirming that *Pf*rh5 was highly conserved in a setting where other vaccine antigens had extensive polymorphism [83,84]

**Figure 4:**
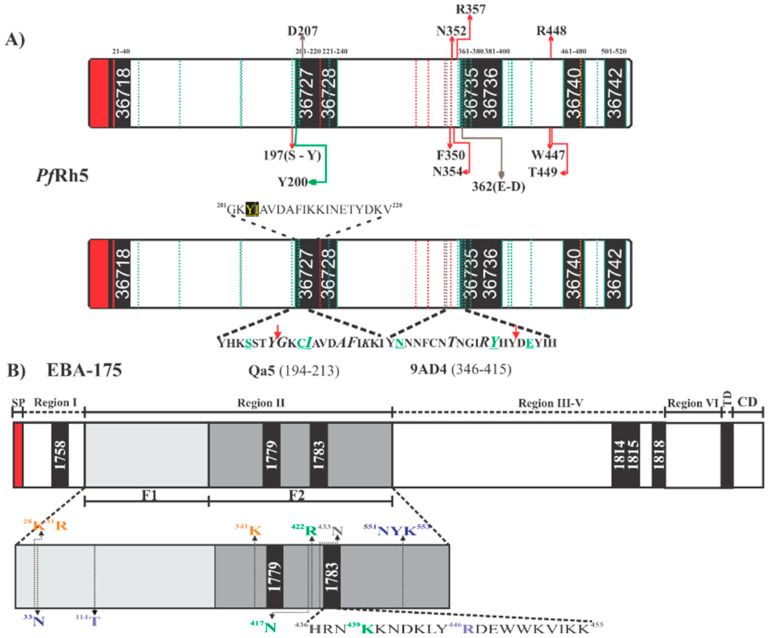
Schematic representation of *Plasmodium falciparum* proteins. (**A**) Scheme for *Pf*Rh5, highlighting High Activity Binding Peptides (HABPs) location and the sequences containing residues involved in binding to BSG (top bar/box), the aforementioned residues having red arrows means *Pf*Rh5 contact sites with the basigin amino terminal extreme. *Pf*Rh5 residues contacting the BSG C-terminal are shown by brown lines and the green line show the residue contacting BSG ^102^H. Green dotted lines indicate sites having polymorphism in *Pf*Rh5; red dotted lines indicate Cys location. The bottom bar/box shows *Pf*Rh5 residues contacting mAbs 9AD4 and QA5. The red arrow indicates the beginning of the sequence for HABP 36727 and 36735 participating in such contact. Residues shown in green are mAbs critical interaction residues. The complete HABP 36727 sequence is shown; residues shown in yellow underlined in black mean polymorphic sites. (**B**) Scheme for EBA-175, highlighting HABP location and the location of each EBA-175 region. The scheme also shows EBA-175 critical residues involved in EBA-175 dimerization (purple), those involved in interaction with glycans 1 and 2 (green), residues contacting glycans 3 and 4 (blue) and residues contacting glycans 5 and 6 (orange). Residues shown in grey are those involved in molecular interactions during complex formation. HABP 1783 (^436^H-K^455^) sequence shows the critical residues participating in each of the aforementioned interactions. TM (transmembrane domain), CD (cytoplasmic domain). Red shows signal peptide location in EBA-175 and *Pf*Rh5.

**Figure 5:**
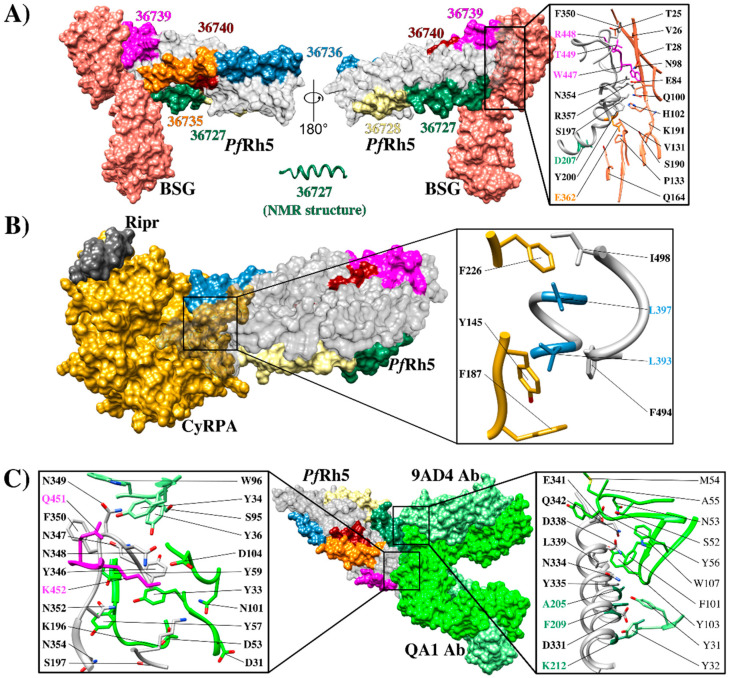
*Pf*Rh5 structure and its interactions (**A**) Structure of the interaction between *Pf*Rh5-BSG (PDB 4U0Q) [74], highlighting HABP binding to RBC: 36727 (green), 36728 (yellow), 36735 (orange), 36736 (blue), and 36740 (brown). HABP 36727 structure (green) determined by nuclear magnetic resonance (NMR), zooming in on residues in the interaction interface; pink shows HABP 36739 residues ^448^R, ^449^T, and ^447^W (close to HABP 36740), green HABP 36727 ^207^D and yellow HABP 36735 ^362^E; (**B**) Crystallographic structure of the Ripr, CyRPA and *Pf*Rh5 ternary complex in contact with the BSG receptor (PDB 6MPV) [76], zooming in on the residues belonging to the interaction interface between *Pf*Rh5-CyRPA, HABP 36736 aa ^393^L, and ^397^L shown in blue; (**C**) Rh5 crystallographic structure, showing points of contact with mAbs QA1 (PDB 4U1G) [74] and 9AD4 (PDB 4U0R) [74] highlighting HABP 36739 residues ^451^Q and ^452^K in pink and HABP residues 36727 ^205^A, ^209^F, and ^212^K in green.

Crystallographic studies have shown that *Pf*Rh5 (residues 140–526 lacking 248–296) (Figure 4A) has a flat rigid structure constituted by two domains, each mainly formed by three helices. The N-terminal domain starts with a short β-sheet, followed by a short α-helix and two large α-helices connected by a truncated loop, while the C-terminal domain is formed by three large α-helices covering the domain’s whole length. There are five cysteines (Cys) throughout its structure; the loop joining the structure’s two domains is stabilized by a disulfide bond (^345^C-C^351^), while another disulfide bond (^224^C-C^317^) joins the second and third helices in the N-terminal domain, leaving a non-bonded Cys (^329^C) (Figure 5A) [74].

*Pf*Rh5 is located in rhoptry necks and is released on merozoite surface to interact (via its N-terminus) with the P113 protein (GPI-anchored to the parasite surface); *Pf*Rh5 interacts also with the soluble Cyrpa protein through its C-terminus, and Cyrpa interacts also with RipR [76,85]. This *Pf*Rh5-mediated protein complex binds to BSG on erythrocyte membrane (Figure 5A) [74,79,86]. BSG is a glycoprotein belonging to the immunoglobulin superfamily. It has two extracellular immunoglobulin domains, a transmembrane region consisting of 23 highly conserved aa, a cytoplasmatic region, and three N-glycosylations [19,87]. These glycosylations do not affect *Pf*Rh5 binding, indicating that this protein’s binding site is only located in the BSG protein core.

BSG and *Pf*Rh5 binding studies have managed to define the hotspots extending between residue ^197^S and ^449^T in *Pf*Rh5 and various contact sites with the amino-terminal domain, C-terminal, and the residue linker ^102^H in BSG (Figure 4A). This receptor binds to the tip of *Pf*Rh5, having a 1,350 Å^2^ contact area where *Pf*Rh5 residues ^350^F and ^447^W stabilize the interaction by packing into hydrophobic pockets on BSG. Interestingly, most SNPs identified in Rh5 have been distributed throughout the protein’s structure; however, they have not affected BSG contact residues [74]. By contrast, some single nucleotide polymorphisms (SNPs) have been associated with an increase in some strains’ ability to invade *Aotus* erythrocytes. Some of them (^204^I, ^347^N, ^358^Y, and ^362^E) are in or close to the BSG binding site and may affect host tropism [88].

Along with structural evidence, fine mapping of *Pf*Rh5 erythrocyte hotspots has led to ascertaining that seven 20 residue-long peptides (called high activity binding peptides - HABPs) specifically interact and have a sub-micromolar affinity with erythrocyte surface. Such HABPs have been located throughout *Pf*Rh5: 36718 (^21^N-T^40^), 36727 (^201^G-V^220^), 36728 (^221^K-H^240^), 36735 (^361^D-L^380^), 36736 (^381^S-K^400^), 36740 (^461^D-Y^480^) and 36742 (^501^L-K^520^) (Figure 4A,B) (33). Some HABP 36727 and 36735 residues overlapped previously reported BSG-*Pf*Rh5 contact residues (Figure 4A and Figure 5A), i.e., HABP 36727 residue ^207^D and HABP 36735 ^362^E/D (Figure 4A and Figure 5A). HABP 36727, having the polymorphic Cys in position 203 replaced by tyrosine, was the only HABP capable of inhibiting invasion by two *P. falciparum* strains (50–80%) [89].

Developing anti-*Pf*Rh5 mAbs for exploring this protein’s importance during invasion (Figure 5C) and its interaction with BSG have led to inconsistent results, i.e., mAbs’ ability to block the *Pf*Rh5-BSG interaction has not correlated with their ability to neutralize and inhibit parasite invasion in some cases. For example, mAb 9AD4 could not inhibit *Pf*Rh5 interaction with its receptor but it was able to neutralize parasite entry to erythrocytes by 70% (3D7 and FVO strains) [32,74]. Mapping epitopes for mAb 9AD4 showed that this mAb recognized the peptide located between residues ^346^YNNNFCNTNGIRYHYDEYIH^364^, partially overlapping HABP 36735 (Figure 4A). However, structural analysis has shown that this mAb also contacts *Pf*Rh5 helices 2 and 3 where residues ^205^A, ^209^F, and ^212^K are located [19], which form part of HABP 36727; this HABP displayed marked ability to block invasion [88]. Despite not having found a relationship between interaction blocking and functional blocking, it is evident that the region in semi-conserved HABP 36727 (^203^C/Y) is a hotspot regarding intervention against *P. falciparum*. Interestingly, it has been found that a single change in this HABP’s aa (position ^204^K/I) has modified the peptide’s 3D structure, thereby resulting in a loss of specific binding to human RBC and its inhibition ability, while binding to *Aotus* RBC has remained unmodified [74,89].

It has been found that mAbs QA1 (38% parasite neutralization), QA5 (63%), and 6BF10 (30%), produced in mice by immunizing with *Pf*Rh5, were capable of blocking BSG interaction [32]. mAb QA1 recognized a conformational epitope directly overlapping the BSG N-terminal domain binding site (Figure 5C) [30,32]. Despite this, inhibition percentages were relatively low compared to those for mAb 9AD4. QA5 inhibitory Ab recognized a linear epitope between residues ^194^YHKSSTYGKCIAVDAFIKKI^213^ overlapping HABP 36727 (underlined), leading to the conclusion that regions or Abs targeting HABP 36727 amino-terminal region are essential for parasite entry regardless of their strong interaction or lack of interaction with BSG (Figure 4A).

Interestingly, Alanine et al., [30] identified two highly neutralizing mAbs (R5.016 and R5.004) against different *P. falciparum* strains, from a panel of mAbs against *Pf*Rh5, isolated from the phase I clinical trial of a *Pf*Rh5-based vaccine [63]. However, although both Abs having EC_50_ values comparable to the most potent anti-merozoite mouse-derived mAbs described to date [32], only Ab R5.004 was able to block basigin interaction. mAb R5.016 (the most potent antibody) could not block interaction with *Pf*Rh5-basigin, *Pf*Rh5-Cyrpa, or *Pf*Rh5-P113. HDX-MS confirmed that R5.004 competes for binding with QA1 and R5.016 with 9AD4. It has been seen that mAb R5.004 has bound *Pf*Rh5 towards the tip of the kite-like structure, contacting helix 4 N-terminus and each of the three loops joining the converging helices at this *Pf*Rh5 vertex. mAb R5.016 has a binding site in *Pf*Rh5 helix 2 N-terminal domain, suggesting that while simultaneous binding is possible, R5.004 binds to a specific epitope having some overlap with that of QA1 and that R5.016 proximity to the BSG binding site could form a steric hindrance mechanism, similar to that speculated for Ab 9AD4 [30].

Growth inhibition tests (GIA) have shown mAb R5.011’s synergistic effect which, despite not having individually neutralizing properties, can boost functional Abs targeting *Pf*Rh5 invasion complex antigens and other targets, such as *Pf*Rh4 and *Pf*AMA1, suggesting a new strategy for designing anti-*P. falciparum* vaccines [30]. However, such results require deeper analysis to enable understanding of the molecular mechanism’s inhibition ability.

It should be considered that *Pf*Rh5’s functional role does not just limit its interaction with BSG; biochemical studies have found that *Pf*Rh5 is tethered to parasite surface by direct interaction via its amino-terminal extreme (a linear sequence of 19 aa (^9^K-K^27^) with membrane-anchored protein P113 [85]. *Pf*Rh5 also forms a ternary complex with *Pf*Rh5-interacting protein (*Pf*Ripr) and cysteine-rich protective antigen (CyRPA) (Figure 5B) [25,76,90,91]. Cryogenic electron microscopy (CryoEM) and biochemical binding studies have found that *Pf*Rh5 is positioned parallel to erythrocyte membrane and that the complex becomes disassembled after binding to BSG and CyRPA (binding to both Rh5 and Ripr). CyRPA becomes excluded from the membrane, while *Pf*Rh5 and Ripr become inserted into it [76], thereby enabling successful merozoite invasion and pore formation on RBC membrane facilitating Ca^2+^ entry to erythroid cells (28). Abs targeting this ternary complex inhibit merozoite invasion, thereby confirming its importance during invasion [90].

It has been found that CyRPA has a monomer 6-bladed β-propeller structure, having four intra-sheet disulfide bonds and one inter-sheet disulfide bond and interacts with Ripr by N-terminal six-stranded β-sheets and *Pf*Rh5 by C-terminal four- and five-stranded β-sheets [76,92,93]. mAb binding to CyRPA is important for characterizing the interaction with *Pf*Rh5. mAb 8A7 has been found to bind to the first and second β-sheets of CyRPA [92] and mAb C12 to the second and third β-sheets [93]. These two block *Pf*Rh5-CyRPA binding but do not overlap the binding epitopes, suggesting that they act by steric hindrance. However, it has been shown that Abs produced during a clinical trial of a *Pf*Rh5-based vaccine blocking *Pf*Rh5-Cyrpa interaction were unable to block merozoite entry to erythrocytes [30]. Although this does not rule out the importance of Abs targeting Cyrpa, it does emphasize *Pf*Rh5 protein relevance in this protein complex since Abs targeting *Pf*Rh5-basigin contact sites and having greater relevance than those targeting HABP 36727 and its neighboring regions, were able to inhibit invasion by up to 70%.

### 3.2. The Main Route: EBA-175-Glycophorin A

**Figure 6:**
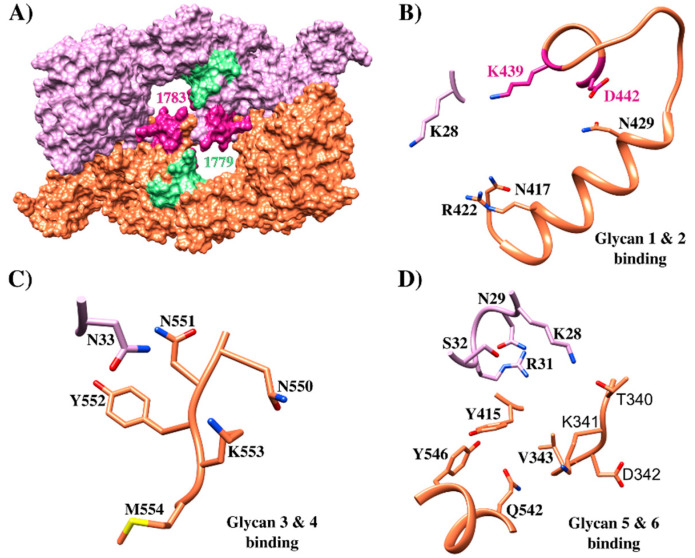
EBA-175 structure and its critical interactions. (**A**) Crystallographic structure of EBA-175 (PDB 1ZRL) [95] highlighting HABPs 1779 (green) and 1783 (pink); (**B**) glycan 1 and 2 binding sites to EBA-175, HABP 1783 ^439^K and ^442^D residues contacting these glycans are highlighted in pink (PDB 1ZRO) [95]; (**C**) glycan 3 and 4 EBA-175 binding sites (PDB 1ZRO) [95] (**D**) glycan 5 and 6 EBA-175 binding (PDB 1ZRO) [95].

EBA-175 protein has been the best-characterized member of the EBL family [12]. It is a transmembrane type 1 protein located in merozoite apical pole micronemes [94] and consists of ~1,505 residues, having a 175 kDa molecular weight. The structure is distributed according to that shared by EBL family members, having a signal sequence followed by six regions (RI–RVI). RII and RVI have highly conserved Cys regions. RII has two DBL domains including the receptor-binding domain and is divided into regions F1 and F2. These six regions are followed by a transmembrane region keeping it anchored to merozoite membrane and a small cytoplasmatic tail (Figure 4B). EBA-175 binds to the host receptor glycophorin A (GPA) (Figure 4B and Figure 6B) [95,96] which is resistant to enzyme treatment with chymotrypsin and susceptible to neuraminidase (cleaves sialic acid ligated to glycophorin’s oligosaccharide α-chains) [97,98,99,100]. The EBA-175-GPA interaction has a well-defined role in anchoring the parasite during erythrocyte invasion moving from merozoite apical end to their posterior end. EBA-175 becomes shed from the parasite surface into the surrounding media during the last steps of invasion [96,101]. EBA-175 alters RBC membrane rigidity and triggers cell signaling to facilitate invasion [94,102].

GPA was the first *P. falciparum* receptor identified in RBC. It is the transmembrane protein expressed with the greatest abundance on the RBC membrane (1 × 10^6^ copies per cell) [32]. It consists of 150 aa (72 in the extracellular domain, followed by 23 in the transmembrane domain, and 36 in the intracellular domain) and has N-glucoside chain in the ^26^N residue [97]. EBA-175 binding to GPA is mediated by Cys-rich RII formed by DBL domains F1 (residues 8–282) and F2 (residues 297–603) (Figure 4B) [95,96]. EBA-175’s F2 domain has received major attention as the region has been shown to bind erythrocytes independently of F1 by contributing to two-third of the residues forming EBA-175 dimer channels [95]. Abs against *Pf*EBA F2 can effectively alter merozoite erythrocyte binding affinity. The RII structure having glycan α-2,3-sialyllactose has been co-crystallized for identifying the EBA-175/GPA interaction site. This glycan contains Neu5Ac(α2,3)-Gal which is required for ligand binding [95], thereby enabling EBA-175 RII recombinant protein to be resolved in a homodimer in which two RII molecules are arranged anti-parallel and interact in a handshake-like manner (Figure 6A). The homodimer center contains two channels; most residues forming the channel’s surface belong to the dimer’s two F2 domains. The dimer’s interface contains six glycan-binding sites, four located inside the channel, and two exposed in a cavity on the external surface. All six glycans contact residues from both RII monomers (Figure 6B–D), indicating that EBA-175 dimerization is biologically important for receptor binding and RBC invasion [95,97,100].

Mutagenesis studies have evaluated residues involved in dimer interactions and glycan-binding. When residue ^446^R (forming a direct salt bridge as well as a water-mediated interaction between monomers) was mutated, EBA175 binding to erythrocyte became reduced. Moreover, steric disruption of interactions with ^114^T (forming van der Waals interactions with ^338^L and ^340^T side chains) by mutation to phenylalanine (T^114^F) led to reduced binding efficiency. ^417^N, ^422^R, and ^439^K mutation led to reduced binding (i.e., residues involved in interaction with glycans 1 and 2) while ^33^N, ^551^N, ^552^Y, and ^553^K mutation (involved in interaction with glycans 3 and 4) reduced binding to different extents. ^28^K, ^31^R, and ^341^K (interacting with glycans 5 and 6) also resulted in varying degrees of reduced binding (Figure 6) [95]. The GPA regions contacting EBA-175 involve sialic acids in the GPA mucin domain, preferentially Neu5Ac(α2-)Gal(Neu5Ac(α-2,6)Gal) on O-linked tetrasaccharides encoded by exon 3. The triple glycan mutant, missing three glycans otherwise attached to ^66^Ser, ^69^Ser, and ^72^Thr, did not bind to EBA-175 RII at all, showing that the three GPA polypeptide fragment glycans encoded by exon 3 are critical for EBA-175 high-avidity binding to GPA on erythrocytes [98,100,103]. Conversely, other studies including flow cytometry and ELISA analysis have provided evidence that the EBA-175 ligand may also bind to desialylated GPA by engaging a region III and IV 21 aa fragment (aa 1076–1096) [104,105]. Peptides comprising aa 1085–1096 contain the erythrocyte binding site and have strongly inhibited parasite invasion [105]. It has been hypothesized that EBA-175 takes part in the second step of two-step erythrocyte binding to explain the presence of other regions [97]. Moreover, RII binding to GPA and Neu5Ac(2–3)Gal showed similar affinity, whereas the full-length EBA-175 ectodomain bound GPA with two-fold higher affinity than glycan alone [31]. These results argued that other EBA-175 extracellular regions outside RII also are involved in interactions with target cells.

However, it should also be considered that RII is highly polymorphic, strong evidence has been advanced for it being under immune selection, thus maintaining genetic diversity in parasite populations [106]. Analyzing genetic variation for identifying the type of selection to which the *eba-175* gene is submitted in three African populations has shown excess intermediate frequency polymorphisms consistent with a hypothesis of balancing selection [107]. Structural analysis, combined with modified calculation of Tajima’s D test, has shown that a large part of the F1 domain appears to be under balancing selection, as is a surface loop in the F2 domain (residues ^432^S - N^442^). The region with the highest calculated spatiality (Tajima’s D test values) was contained within the RII F1 domain, predominantly consisting of residues ^266^E-D^289^, ^314^P-Q^322^, and ^382^L-L^400^ [108]. It has been well established that the gene encoding EBA-175 has a highly divergent dimorphic segment of sequences in region III. The first was detected in the FCR3 strain, called the F loop (342 bp insertion), and the second was identified in the CAMP strain, called the C loop (423 bp insertion) [109]. F/C segment relevance is not well known but parts of them have been shown to vary amongst natural parasite populations in Africa [110,111]. This might explain the lack of association with protective immunity in cohort studies which would depend on the allele type used for detecting Ab responses, posing a major hurdle to its consideration as an effective vaccine candidate. EBA-175 RIII–V-specific Abs have also been associated with protection from symptomatic malaria [112]. Moreover, a previous study has shown that immunization with recombinant RIII–V induces Abs potently inhibiting merozoite invasion [105]. Testing Abs raised against two EBA-175 RIII-V dimorphic alleles indicated that dimorphic region III contributed little to vaccine-induced immunity and conserved RIV-V contained inhibitory Abs’ predominant epitopes [105].

Six HABPs distributed throughout the whole of EBA-175 were identified when it was synthesized in sequential 20 non-overlapping aa peptides (Figure 4B). Peptide 1758 (^80^K-N^99^) was located in RI for which no binding activity has been reported before, peptides 1779 (^356^N-I^375^) and 1783 (^436^H-K^455^) were located in RII (sub-region denoted as 5’Cys F2) previously reported as being a binding region and 1815 (^1220^Y-H^1239^) and 1818 (^1280^N-L^1299^) were located in RIV (Figure 4B and Figure 5A). In-depth analysis showed that ^436^H enabled dimerization with a contralateral ^435^V (both residues contained in HABP 1783) in an inverted EBA-175 molecule, forming a handshake-like structure. Residues ^439^K and ^442^D bound glycophorin A glycan 5 Neu5Ac1 via H-bonds. HABP 1783 ^450^W has also induced fold stabilization at this site in the same RII and contained critical residue ^446^R establishing a salt bridge with ^30^D, thereby dramatically reducing RBC binding when ^446^R is mutated (Figure 4B and Figure 6) [95,113,114].

Similarly, HABP 1779 has been localized close to glycan 5 and 6 binding sites in the F1 region (Figure 6D) but did not interact directly with them [99,100], HABP 1815 (Y^1220^-H^1239^) has been seen to contain a conserved sequence in all EBL molecules in RV, whereas HABP 1818 RV residues R^1258^ and F^1265^ bound to mediate EBA-175 traffic from merozoite micronemes to their membrane [114,115]. HABP 1818 has been located close to rhomboid protease 4 cleavage site enabling EBA-175 release during invasion for parasitophorous vacuole (PV) formation [114]. All small pieces of peptides covering the whole protein take part in relevant functions during invasion, such as binding, traffic, and cleavage site.

It has been shown that anti-EBA-175 RII Abs have potently blocked native EBA-175 binding to erythrocytes and even inhibited *P. falciparum* strain invasion of erythrocytes by pathways that do not require sialic acid for invasion in vitro [116,117] EBA-175 RII function has been evaluated by creating panels of mAbs derived from animal models. mAbs R215, R217, and R256 are specific and compete for the F2 RII domain, potently blocking EBA-175 binding to RBCs and merozoite invasion of RBC. However, mAb R217 had 100-fold lower IC_50_ than R218 against parasites in culture. Structure determination has revealed that this mAb targeted a conformational epitope containing residues 475–485 and 561–567 overlapping the dimer interface and glycan-binding residues. mAb R216 has recognized F2 to a reduced extent but could not effectively block EBA-175 binding to RBC; mAb R218 has recognized the F1 domain, managing to inhibit parasite growth in a less efficient manner than F2-specific Abs [118].

All these known results for EBA-175 highlight the correlation between the interaction interface involving HABP 1783 and neutralizing epitopes. However, such analysis must be enriched with Abs produced upon immunization with other EBA-175 regions having limited polymorphism, such as the conserved RIV-V region where predominant inhibitory Ab epitopes have been reported [105]. It cannot be ruled out that regions outside RII could be strongly involved in RBC interaction [119]. Studies involving synthetic peptides have shown that three of them located in regions IV and V (Figure 4B) interact strongly with RBC.

## 4. Conclusions

*P. falciparum* and *P. vivax* have multiple, complex RBC invasion routes mediated by specific receptor-ligand interactions. Elucidating and combining functional studies with structural ones enables contributing to the development of prophylactic and/or therapeutic measures helping to mitigate malaria, a disease posing a drastic worldwide public health problem. The proteins analyzed in this review impose great challenges for studying them in-depth due to their complexity concerning their high polymorphism (DBP1 and EBA175) or participation in molecular complex formation (*Pf*Rh5). Analyzing these important proteins has revealed hotspots (small regions) which might be converted into important intervention points, i.e., DBP1 HARPB 1639 located in SD3, *Pf*Rh5 HABP 36727 located in a different site than that for contact with its main BSG receptor and HABP 1783 located in the EBA-175’s F2 region, directly contacting GPA. However, it should be stressed regarding EBA-175 that the search should also be directed towards other regions, such as IV and V (having functional evidence of binding), for resolving the high level of polymorphism detected in region II.

## Figures and Tables

**Table 1:** *Plasmodium vivax* blood stage malaria vaccine candidates.

Vaccine Candidate	Development Phase	Antigen	Formulation	Ref
*Pv*DBPII-DEK^null^	Pre-clinical	DBP1	Rec. protein- adjuvant	[66]
*Pv*MSP1_19_	Pre-clinical	*Pv*MSP1	Rec. protein-Montanide ISA720	[67,68]
ChAd63-*Pv*AMA1/MVA-*PvA*MA1	Pre-clinical	*Pv*AMA1	Prime boost, viral vectors	[69]
*Pv*AMA1	Pre-clinical	*Pv*AMA1	Rec. protein-adjuvant	[70]
ChAd63-*Pv*DBPII/MVA-*Pv*DBPII	Clinical Phase Ia	DBP1	Prime boost, viral vectors	[63]
*Pv*DBPII/GLA-SE	Clinical Phase Ia	DBP1	Rec. protein-GLA-SE	[62]
*Pv*DBPII/Matrix M1	Clinical Phase I/IIa	DBP1	Rec. protein-adjuvant	N.A.

Rec. protein: Recombinant protein; N.A.: Not available.

## Supplementary Materials

The following are available online https://www.mdpi.com/1422-0067/21/13/4729/s1, **Table S1**: The main ligand-receptor interactions taking place during merozoite invasion of RBCs or reticulocytes.

## Abbreviations

bnAb	broadly neutralizing antibody
BSG	basigin
CryoEM	cryogenic electron microscopy
CyRPA	cysteine-rich protective antigen
Cys	cysteine
DARC	Duffy antigen receptor for chemokines
DBL	Duffy binding-like
DBP1	Duffy binding protein 1
EBA175	erythrocyte binding antigen 175
GPA	glycophorin A
HABP	high activity binding peptide
HARBP	high activity reticulocyte binding peptide
IPP	protein-protein interaction
ITC	isothermal titration calorimetry
mAb	monoclonal antibody
NMR	nuclear magnetic resonance
*Pf*Rh5	*Plasmodium falciparum* reticulocyte-binding protein homologue 5
PV	parasitophorous vacuole
RBC	red blood cell
RII-DBP	region II Duffy binding protein
Ripr	Rh5-interacting protein
SD1	subdomain 1
SD2	subdomain 2
SD3	subdomain 3
TJ	tight junction
TfR-1	transferrin receptor 1

## References

[1] Phillips M.A., Burrows J.N., Manyando C., van Huijsduijnen R.H., Van Voorhis W.C., Wells T.N.C. (2017). Malaria. Nat. Rev. Dis. Primers.

[2] Florens L., Washburn M.P., Raine J.D., Anthony R.M., Grainger M., Haynes J.D., Moch J.K., Muster N., Sacci J.B., Tabb D.L. (2002). A proteomic view of the Plasmodium falciparum life cycle. Nature.

[3] Bozdech Z., Llinas M., Pulliam B.L., Wong E.D., Zhu J., DeRisi J.L. (2003). The transcriptome of the intraerythrocytic developmental cycle of Plasmodium falciparum. PLoS Biol..

[4] Miller L.H., Baruch D.I., Marsh K., Doumbo O.K. (2002). The pathogenic basis of malaria. Nature.

[5] White N.J., Pukrittayakamee S., Hien T.T., Faiz M.A., Mokuolu O.A., Dondorp A.M. (2014). Malaria. Lancet.

[6] Tonkin M.L., Boulanger M.J. (2015). The shear stress of host cell invasion: Exploring the role of biomolecular complexes. PLoS Pathog..

[7] Weiss G.E., Crabb B.S., Gilson P.R. (2016). Overlaying Molecular and Temporal Aspects of Malaria Parasite Invasion. Trends Parasitol..

[8] Gaur D., Mayer D.C., Miller L.H. (2004). Parasite ligand-host receptor interactions during invasion of erythrocytes by Plasmodium merozoites. Int. J. Parasitol..

[9] Rayner J.C., Galinski M.R., Ingravallo P., Barnwell J.W. (2000). Two Plasmodium falciparum genes express merozoite proteins that are related to Plasmodium vivax and Plasmodium yoelii adhesive proteins involved in host cell selection and invasion. Proc. Natl. Acad. Sci. USA.

[10] Gunalan K., Gao X., Yap S.S., Huang X., Preiser P.R. (2013). The role of the reticulocyte-binding-like protein homologues of Plasmodium in erythrocyte sensing and invasion. Cell. Microbiol..

[11] Gilson P.R., Crabb B.S. (2009). Morphology and kinetics of the three distinct phases of red blood cell invasion by Plasmodium falciparum merozoites. Int. J. Parasitol..

[12] Tham W.H., Healer J., Cowman A.F. (2012). Erythrocyte and reticulocyte binding-like proteins of Plasmodium falciparum. Trends Parasitol..

[13] Riglar D.T., Richard D., Wilson D.W., Boyle M.J., Dekiwadia C., Turnbull L., Angrisano F., Marapana D.S., Rogers K.L., Whitchurch C.B. (2011). Super-resolution dissection of coordinated events during malaria parasite invasion of the human erythrocyte. Cell Host Microbe.

[14] Dvorak J.A., Miller L.H., Whitehouse W.C., Shiroishi T. (1975). Invasion of erythrocytes by malaria merozoites. Science.

[15] Boyle M.J., Richards J.S., Gilson P.R., Chai W., Beeson J.G. (2010). Interactions with heparin-like molecules during erythrocyte invasion by Plasmodium falciparum merozoites. Blood.

[16] Weiss G.E., Gilson P.R., Taechalertpaisarn T., Tham W.H., de Jong N.W., Harvey K.L., Fowkes F.J., Barlow P.N., Rayner J.C., Wright G.J. (2015). Revealing the sequence and resulting cellular morphology of receptor-ligand interactions during Plasmodium falciparum invasion of erythrocytes. PLoS Pathog..

[17] Lopaticki S., Maier A.G., Thompson J., Wilson D.W., Tham W.H., Triglia T., Gout A., Speed T.P., Beeson J.G., Healer J. (2011). Reticulocyte and erythrocyte binding-like proteins function cooperatively in invasion of human erythrocytes by malaria parasites. Infect. Immun..

[18] Stubbs J., Simpson K.M., Triglia T., Plouffe D., Tonkin C.J., Duraisingh M.T., Maier A.G., Winzeler E.A., Cowman A.F. (2005). Molecular mechanism for switching of P. falciparum invasion pathways into human erythrocytes. Science.

[19] Crosnier C., Bustamante L.Y., Bartholdson S.J., Bei A.K., Theron M., Uchikawa M., Mboup S., Ndir O., Kwiatkowski D.P., Duraisingh M.T. (2011). Basigin is a receptor essential for erythrocyte invasion by Plasmodium falciparum. Nature.

[20] Aikawa M., Miller L.H., Johnson J., Rabbege J. (1978). Erythrocyte entry by malarial parasites. A moving junction between erythrocyte and parasite. J. Cell Biol..

[21] Besteiro S., Michelin A., Poncet J., Dubremetz J.F., Lebrun M. (2009). Export of a Toxoplasma gondii rhoptry neck protein complex at the host cell membrane to form the moving junction during invasion. PLoS Pathog..

[22] Srinivasan P., Beatty W.L., Diouf A., Herrera R., Ambroggio X., Moch J.K., Tyler J.S., Narum D.L., Pierce S.K., Boothroyd J.C. (2011). Binding of Plasmodium merozoite proteins RON2 and AMA1 triggers commitment to invasion. Proc. Natl. Acad. Sci. USA.

[23] Lin C.S., Uboldi A.D., Epp C., Bujard H., Tsuboi T., Czabotar P.E., Cowman A.F. (2016). Multiple Plasmodium falciparum Merozoite Surface Protein 1 Complexes Mediate Merozoite Binding to Human Erythrocytes. J. Biol. Chem..

[24] Ranjan R., Chugh M., Kumar S., Singh S., Kanodia S., Hossain M.J., Korde R., Grover A., Dhawan S., Chauhan V.S. (2011). Proteome analysis reveals a large merozoite surface protein-1 associated complex on the Plasmodium falciparum merozoite surface. J. Proteome Res..

[25] Reddy K.S., Amlabu E., Pandey A.K., Mitra P., Chauhan V.S., Gaur D. (2015). Multiprotein complex between the GPI-anchored CyRPA with *Pf*RH5 and *Pf*Ripr is crucial for Plasmodium falciparum erythrocyte invasion. Proc. Natl. Acad. Sci. USA.

[26] Li X., Chen H., Oo T.H., Daly T.M., Bergman L.W., Liu S.C., Chishti A.H., Oh S.S. (2004). A co-ligand complex anchors Plasmodium falciparum merozoites to the erythrocyte invasion receptor band 3. J. Biol. Chem..

[27] Baldwin M.R., Li X., Hanada T., Liu S.C., Chishti A.H. (2015). Merozoite surface protein 1 recognition of host glycophorin A mediates malaria parasite invasion of red blood cells. Blood.

[28] Urusova D., Carias L., Huang Y., Nicolete V.C., Popovici J., Roesch C., Salinas N.D., Witkowski B., Ferreira M.U., Adams J.H. (2019). Structural basis for neutralization of Plasmodium vivax by naturally acquired human antibodies that target DBP. Nat. Microbiol..

[29] Batchelor J.D., Malpede B.M., Omattage N.S., DeKoster G.T., Henzler-Wildman K.A., Tolia N.H. (2014). Red blood cell invasion by Plasmodium vivax: Structural basis for DBP engagement of DARC. PLoS Pathog..

[30] Alanine D.G.W., Quinkert D., Kumarasingha R., Mehmood S., Donnellan F.R., Minkah N.K., Dadonaite B., Diouf A., Galaway F., Silk S.E. (2019). Human Antibodies that Slow Erythrocyte Invasion Potentiate Malaria-Neutralizing Antibodies. Cell.

[31] Chen E., Paing M.M., Salinas N., Sim B.K., Tolia N.H. (2013). Structural and functional basis for inhibition of erythrocyte invasion by antibodies that target Plasmodium falciparum EBA-175. PLoS Pathog..

[32] Douglas A.D., Williams A.R., KnuePfer E., Illingworth J.J., Furze J.M., Crosnier C., Choudhary P., Bustamante L.Y., Zakutansky S.E., Awuah D.K. (2014). Neutralization of Plasmodium falciparum merozoites by antibodies against *Pf*RH5. J. Immunol..

[33] Rawlinson T.A., Barber N.M., Mohring F., Cho J.S., Kosaisavee V., Gerard S.F., Alanine D.G.W., Labbe G.M., Elias S.C., Silk S.E. (2019). Structural basis for inhibition of Plasmodium vivax invasion by a broadly neutralizing vaccine-induced human antibody. Nat. Microbiol..

[34] World Health Organization (2018). Word Malaria Report 2018.

[35] Cowman A.F., Crabb B.S. (2006). Invasion of red blood cells by malaria parasites. Cell.

[36] Adams J.H., Mueller I. (2017). The Biology of Plasmodium vivax. Cold Spring Harb. Perspect. Med..

[37] Malleret B., Li A., Zhang R., Tan K.S., Suwanarusk R., Claser C., Cho J.S., Koh E.G., Chu C.S., Pukrittayakamee S. (2015). Plasmodium vivax: Restricted tropism and rapid remodeling of CD71-positive reticulocytes. Blood.

[38] Gruszczyk J., Kanjee U., Chan L.J., Menant S., Malleret B., Lim N.T.Y., Schmidt C.Q., Mok Y.F., Lin K.M., Pearson R.D. (2018). Transferrin receptor 1 is a reticulocyte-specific receptor for Plasmodium vivax. Science.

[39] Miller L.H., Mason S.J., Dvorak J.A., McGinniss M.H., Rothman I.K. (1975). Erythrocyte receptors for (Plasmodium knowlesi) malaria: Duffy blood group determinants. Science.

[40] Gunalan K., Lo E., Hostetler J.B., Yewhalaw D., Mu J., Neafsey D.E., Yan G., Miller L.H. (2016). Role of Plasmodium vivax Duffy-binding protein 1 in invasion of Duffy-null Africans. Proc. Natl. Acad. Sci. USA.

[41] Chitnis C.E., Chaudhuri A., Horuk R., Pogo A.O., Miller L.H. (1996). The domain on the Duffy blood group antigen for binding Plasmodium vivax and P. knowlesi malarial parasites to erythrocytes. J. Exp. Med..

[42] Chitnis C.E., Miller L.H. (1994). Identification of the erythrocyte binding domains of Plasmodium vivax and Plasmodium knowlesi proteins involved in erythrocyte invasion. J. Exp. Med..

[43] Hans D., Pattnaik P., Bhattacharyya A., Shakri A.R., Yazdani S.S., Sharma M., Choe H., Farzan M., Chitnis C.E. (2005). Mapping binding residues in the Plasmodium vivax domain that binds Duffy antigen during red cell invasion. Mol. Microbiol..

[44] Batchelor J.D., Zahm J.A., Tolia N.H. (2011). Dimerization of Plasmodium vivax DBP is induced upon receptor binding and drives recognition of DARC. Nat. Struct. Mol. Biol..

[45] Chootong P., Ntumngia F.B., VanBuskirk K.M., Xainli J., Cole-Tobian J.L., Campbell C.O., Fraser T.S., King C.L., Adams J.H. (2010). Mapping epitopes of the Plasmodium vivax Duffy binding protein with naturally acquired inhibitory antibodies. Infect. Immun..

[46] Ampudia E., Patarroyo M.A., Patarroyo M.E., Murillo L.A. (1996). Genetic polymorphism of the Duffy receptor binding domain of Plasmodium vivax in Colombian wild isolates. Mol. Biochem. Parasitol..

[47] Sousa T.N., Ceravolo I.P., Fernandes Fontes C.J., Couto A., Carvalho L.H., Brito C.F. (2006). The pattern of major polymorphisms in the Duffy binding protein ligand domain among Plasmodium vivax isolates from the Brazilian Amazon area. Mol. Biochem. Parasitol..

[48] Xainli J., Adams J.H., King C.L. (2000). The erythrocyte binding motif of plasmodium vivax duffy binding protein is highly polymorphic and functionally conserved in isolates from Papua New Guinea. Mol. Biochem. Parasitol..

[49] Nobrega de Sousa T., Carvalho L.H., Alves de Brito C.F. (2011). Worldwide genetic variability of the Duffy binding protein: Insights into Plasmodium vivax vaccine development. PLoS ONE.

[50] Ntumngia F.B., Schloegel J., Barnes S.J., McHenry A.M., Singh S., King C.L., Adams J.H. (2012). Conserved and variant epitopes of Plasmodium vivax Duffy binding protein as targets of inhibitory monoclonal antibodies. Infect. Immun..

[51] Cole-Tobian J.L., Cortes A., Baisor M., Kastens W., Xainli J., Bockarie M., Adams J.H., King C.L. (2002). Age-acquired immunity to a Plasmodium vivax invasion ligand, the duffy binding protein. J. Infect. Dis..

[52] Tsuboi T., Kappe S.H., al-Yaman F., Prickett M.D., Alpers M., Adams J.H. (1994). Natural variation within the principal adhesion domain of the Plasmodium vivax duffy binding protein. Infect. Immun..

[53] VanBuskirk K.M., Cole-Tobian J.L., Baisor M., Sevova E.S., Bockarie M., King C.L., Adams J.H. (2004). Antigenic drift in the ligand domain of Plasmodium vivax duffy binding protein confers resistance to inhibitory antibodies. J. Infect. Dis..

[54] Singh S.K., Singh A.P., Pandey S., Yazdani S.S., Chitnis C.E., Sharma A. (2003). Definition of structural elements in Plasmodium vivax and P. knowlesi Duffy-binding domains necessary for erythrocyte invasion. Biochem. J..

[55] George M.T., Schloegel J.L., Ntumngia F.B., Barnes S.J., King C.L., Casey J.L., Foley M., Adams J.H. (2019). Identification of an Immunogenic Broadly Inhibitory Surface Epitope of the Plasmodium vivax Duffy Binding Protein Ligand Domain. mSphere.

[56] Ocampo M., Vera R., Eduardo Rodriguez L., Curtidor H., Urquiza M., Suarez J., Garcia J., Puentes A., Lopez R., Trujillo M. (2002). Plasmodium vivax Duffy binding protein peptides specifically bind to reticulocytes. Peptides.

[57] Chen E., Salinas N.D., Huang Y., Ntumngia F., Plasencia M.D., Gross M.L., Adams J.H., Tolia N.H. (2016). Broadly neutralizing epitopes in the Plasmodium vivax vaccine candidate Duffy Binding Protein. Proc. Natl. Acad. Sci. USA.

[58] Guy A.J., Irani V., Richards J.S., Ramsland P.A. (2018). Structural patterns of selection and diversity for Plasmodium vivax antigens DBP and AMA1. Malar. J..

[59] Bourgard C., Albrecht L., Kayano A., Sunnerhagen P., Costa F.T.M. (2018). Plasmodium vivax Biology: Insights Provided by Genomics, Transcriptomics and Proteomics. Front. Cell. Infect. Microbiol..

[60] Chan L.J., Dietrich M.H., Nguitragool W., Tham W.H. (2020). Plasmodium vivax Reticulocyte Binding Proteins for invasion into reticulocytes. Cell. Microbiol..

[61] Auburn S., Getachew S., Pearson R.D., Amato R., Miotto O., Trimarsanto H., Zhu S.J., Rumaseb A., Marfurt J., Noviyanti R. (2019). Genomic Analysis of Plasmodium vivax in Southern Ethiopia Reveals Selective Pressures in Multiple Parasite Mechanisms. J. Infect. Dis..

[62] Singh K., Mukherjee P., Shakri A.R., Singh A., Pandey G., Bakshi M., Uppal G., Jena R., Rawat A., Kumar P. (2018). Malaria vaccine candidate based on Duffy-binding protein elicits strain transcending functional antibodies in a Phase I trial. NPJ Vaccines.

[63] Payne R.O., Silk S.E., Elias S.C., Milne K.H., Rawlinson T.A., Llewellyn D., Shakri A.R., Jin J., Labbe G.M., Edwards N.J. (2017). Human vaccination against Plasmodium vivax Duffy-binding protein induces strain-transcending antibodies. JCI Insight.

[64] King C.L., Michon P., Shakri A.R., Marcotty A., Stanisic D., Zimmerman P.A., Cole-Tobian J.L., Mueller I., Chitnis C.E. (2008). Naturally acquired Duffy-binding protein-specific binding inhibitory antibodies confer protection from blood-stage Plasmodium vivax infection. Proc. Natl. Acad. Sci. USA.

[65] Nicolete V.C., Frischmann S., Barbosa S., King C.L., Ferreira M.U. (2016). Naturally Acquired Binding-Inhibitory Antibodies to Plasmodium vivax Duffy Binding Protein and Clinical Immunity to Malaria in Rural Amazonians. J. Infect. Dis..

[66] Ntumngia F.B., Adams J.H. (2012). Design and immunogenicity of a novel synthetic antigen based on the ligand domain of the Plasmodium vivax duffy binding protein. Clin. Vaccine Immunol..

[67] Rosa D.S., Iwai L.K., Tzelepis F., Bargieri D.Y., Medeiros M.A., Soares I.S., Sidney J., Sette A., Kalil J., Mello L.E. (2006). Immunogenicity of a recombinant protein containing the Plasmodium vivax vaccine candidate MSP1(19) and two human CD4+ T-cell epitopes administered to non-human primates (Callithrix jacchus jacchus). Microbes Infect..

[68] Devi Y.S., Mukherjee P., Yazdani S.S., Shakri A.R., Mazumdar S., Pandey S., Chitnis C.E., Chauhan V.S. (2007). Immunogenicity of Plasmodium vivax combination subunit vaccine formulated with human compatible adjuvants in mice. Vaccine.

[69] Bouillet L.E., Dias M.O., Dorigo N.A., Moura A.D., Russell B., Nosten F., Renia L., Braga E.M., Gazzinelli R.T., Rodrigues M.M. (2011). Long-term humoral and cellular immune responses elicited by a heterologous Plasmodium vivax apical membrane antigen 1 protein prime/adenovirus boost immunization protocol. Infect. Immun..

[70] Vicentin E.C., Francoso K.S., Rocha M.V., Iourtov D., Dos Santos F.L., Kubrusly F.S., Sakauchi M.A., Raw I., Nosten F., Renia L. (2014). Invasion-inhibitory antibodies elicited by immunization with Plasmodium vivax apical membrane antigen-1 expressed in Pichia pastoris yeast. Infect. Immun..

[71] Cowman A.F., Healer J., Marapana D., Marsh K. (2016). Malaria: Biology and Disease. Cell.

[72] Satchwell T.J. (2016). Erythrocyte invasion receptors for Plasmodium falciparum: New and old. Transfus. Med..

[73] Wahlgren M., Goel S., Akhouri R.R. (2017). Variant surface antigens of Plasmodium falciparum and their roles in severe malaria. Nat. Rev. Microbiol..

[74] Wright K.E., Hjerrild K.A., Bartlett J., Douglas A.D., Jin J., Brown R.E., Illingworth J.J., Ashfield R., Clemmensen S.B., de Jongh W.A. (2014). Structure of malaria invasion protein RH5 with erythrocyte basigin and blocking antibodies. Nature.

[75] Adams J.H., Blair P.L., Kaneko O., Peterson D.S. (2001). An expanding ebl family of Plasmodium falciparum. Trends Parasitol..

[76] Wong W., Huang R., Menant S., Hong C., Sandow J.J., Birkinshaw R.W., Healer J., Hodder A.N., Kanjee U., Tonkin C.J. (2019). Structure of Plasmodium falciparum Rh5-CyRPA-Ripr invasion complex. Nature.

[77] Volz J.C., Yap A., Sisquella X., Thompson J.K., Lim N.T., Whitehead L.W., Chen L., Lampe M., Tham W.H., Wilson D. (2016). Essential Role of the *Pf*Rh5/*Pf*Ripr/CyRPA Complex during Plasmodium falciparum Invasion of Erythrocytes. Cell Host Microbe.

[78] Li H., Bogyo M., da Fonseca P.C. (2016). The cryo-EM structure of the Plasmodium falciparum 20S proteasome and its use in the fight against malaria. FEBS J..

[79] Baum J., Chen L., Healer J., Lopaticki S., Boyle M., Triglia T., Ehlgen F., Ralph S.A., Beeson J.G., Cowman A.F. (2009). Reticulocyte-binding protein homologue 5 - an essential adhesin involved in invasion of human erythrocytes by Plasmodium falciparum. Int. J. Parasitol..

[80] Hayton K., Gaur D., Liu A., Takahashi J., Henschen B., Singh S., Lambert L., Furuya T., Bouttenot R., Doll M. (2008). Erythrocyte binding protein *Pf*RH5 polymorphisms determine species-specific pathways of Plasmodium falciparum invasion. Cell Host Microbe.

[81] Bustamante L.Y., Bartholdson S.J., Crosnier C., Campos M.G., Wanaguru M., Nguon C., Kwiatkowski D.P., Wright G.J., Rayner J.C. (2013). A full-length recombinant Plasmodium falciparum *Pf*RH5 protein induces inhibitory antibodies that are effective across common *Pf*RH5 genetic variants. Vaccine.

[82] Manske M., Miotto O., Campino S., Auburn S., Almagro-Garcia J., Maslen G., O’Brien J., Djimde A., Doumbo O., Zongo I. (2012). Analysis of Plasmodium falciparum diversity in natural infections by deep sequencing. Nature.

[83] Ouattara A., Tran T.M., Doumbo S., Adams M., Agrawal S., Niangaly A., Nelson-Owens S., Doumtabe D., Tolo Y., Ongoiba A. (2018). Extent and Dynamics of Polymorphism in the Malaria Vaccine Candidate Plasmodium falciparum Reticulocyte-Binding Protein Homologue-5 in Kalifabougou, Mali. Am. J. Trop. Med. Hyg..

[84] Ajibaye O., Osuntoki A.A., Balogun E.O., Olukosi Y.A., Iwalokun B.A., Oyebola K.M., Hikosaka K., Watanabe Y.I., Ebiloma G.U., Kita K. (2020). Genetic polymorphisms in malaria vaccine candidate Plasmodium falciparum reticulocyte-binding protein homologue-5 among populations in Lagos, Nigeria. Malar. J..

[85] Galaway F., Drought L.G., Fala M., Cross N., Kemp A.C., Rayner J.C., Wright G.J. (2017). P113 is a merozoite surface protein that binds the N terminus of Plasmodium falciparum RH5. Nat. Commun..

[86] Rodriguez M., Lustigman S., Montero E., Oksov Y., Lobo C.A. (2008). *Pf*RH5: A novel reticulocyte-binding family homolog of plasmodium falciparum that binds to the erythrocyte, and an investigation of its receptor. PLoS ONE.

[87] Heller M., von der Ohe M., Kleene R., Mohajeri M.H., Schachner M. (2003). The immunoglobulin-superfamily molecule basigin is a binding protein for oligomannosidic carbohydrates: An anti-idiotypic approach. J. Neurochem..

[88] Galaway F., Yu R., Constantinou A., Prugnolle F., Wright G.J. (2019). Resurrection of the ancestral RH5 invasion ligand provides a molecular explanation for the origin of P. falciparum malaria in humans. PLoS Biol..

[89] Arevalo-Pinzon G., Curtidor H., Munoz M., Patarroyo M.A., Bermudez A., Patarroyo M.E. (2012). A single amino acid change in the Plasmodium falciparum RH5 (*Pf*RH5) human RBC binding sequence modifies its structure and determines species-specific binding activity. Vaccine.

[90] Chen L., Lopaticki S., Riglar D.T., Dekiwadia C., Uboldi A.D., Tham W.H., O’Neill M.T., Richard D., Baum J., Ralph S.A. (2011). An EGF-like protein forms a complex with *Pf*Rh5 and is required for invasion of human erythrocytes by Plasmodium falciparum. PLoS Pathog..

[91] Ntege E.H., Arisue N., Ito D., Hasegawa T., Palacpac N.M.Q., Egwang T.G., Horii T., Takashima E., Tsuboi T. (2016). Identification of Plasmodium falciparum reticulocyte binding protein homologue 5-interacting protein, *Pf*Ripr, as a highly conserved blood-stage malaria vaccine candidate. Vaccine.

[92] Chen L., Xu Y., Wong W., Thompson J.K., Healer J., Goddard-Borger E.D., Lawrence M.C., Cowman A.F. (2017). Structural basis for inhibition of erythrocyte invasion by antibodies to Plasmodium falciparum protein CyRPA. Elife.

[93] Favuzza P., Guffart E., Tamborrini M., Scherer B., Dreyer A.M., Rufer A.C., Erny J., Hoernschemeyer J., Thoma R., Schmid G. (2017). Structure of the malaria vaccine candidate antigen CyRPA and its complex with a parasite invasion inhibitory antibody. Elife.

[94] Koch M., Wright K.E., Otto O., Herbig M., Salinas N.D., Tolia N.H., Satchwell T.J., Guck J., Brooks N.J., Baum J. (2017). Plasmodium falciparum erythrocyte-binding antigen 175 triggers a biophysical change in the red blood cell that facilitates invasion. Proc. Natl. Acad. Sci. USA.

[95] Tolia N.H., Enemark E.J., Sim B.K., Joshua-Tor L. (2005). Structural basis for the EBA-175 erythrocyte invasion pathway of the malaria parasite Plasmodium falciparum. Cell.

[96] Duraisingh M.T., Maier A.G., Triglia T., Cowman A.F. (2003). Erythrocyte-binding antigen 175 mediates invasion in Plasmodium falciparum utilizing sialic acid-dependent and -independent pathways. Proc. Natl. Acad. Sci. USA.

[97] Jaskiewicz E., Jodlowska M., Kaczmarek R., Zerka A. (2019). Erythrocyte glycophorins as receptors for Plasmodium merozoites. Parasites Vectors.

[98] Orlandi P.A., Klotz F.W., Haynes J.D. (1992). A malaria invasion receptor, the 175-kilodalton erythrocyte binding antigen of Plasmodium falciparum recognizes the terminal Neu5Ac(alpha 2-3)Gal- sequences of glycophorin A. J. Cell Biol..

[99] Wanaguru M., Crosnier C., Johnson S., Rayner J.C., Wright G.J. (2013). Biochemical analysis of the Plasmodium falciparum erythrocyte-binding antigen-175 (EBA175)-glycophorin-A interaction: Implications for vaccine design. J. Biol. Chem..

[100] Salinas N.D., Paing M.M., Tolia N.H. (2014). Critical glycosylated residues in exon three of erythrocyte glycophorin A engage Plasmodium falciparum EBA-175 and define receptor specificity. MBio.

[101] O’Donnell R.A., Hackett F., Howell S.A., Treeck M., Struck N., Krnajski Z., Withers-Martinez C., Gilberger T.W., Blackman M.J. (2006). Intramembrane proteolysis mediates shedding of a key adhesin during erythrocyte invasion by the malaria parasite. J. Cell Biol..

[102] Sisquella X., Nebl T., Thompson J.K., Whitehead L., Malpede B.M., Salinas N.D., Rogers K., Tolia N.H., Fleig A., O’Neill J. (2017). Plasmodium falciparum ligand binding to erythrocytes induce alterations in deformability essential for invasion. Elife.

[103] Sim B.K., Chitnis C.E., Wasniowska K., Hadley T.J., Miller L.H. (1994). Receptor and ligand domains for invasion of erythrocytes by Plasmodium falciparum. Science.

[104] Jakobsen P.H., Heegaard P.M., Koch C., Wasniowska K., Lemnge M.M., Jensen J.B., Sim B.K. (1998). Identification of an erythrocyte binding peptide from the erythrocyte binding antigen, EBA-175, which blocks parasite multiplication and induces peptide-blocking antibodies. Infect. Immun..

[105] Healer J., Thompson J.K., Riglar D.T., Wilson D.W., Chiu Y.H., Miura K., Chen L., Hodder A.N., Long C.A., Hansen D.S. (2013). Vaccination with conserved regions of erythrocyte-binding antigens induces neutralizing antibodies against multiple strains of Plasmodium falciparum. PLoS ONE.

[106] Baum J., Thomas A.W., Conway D.J. (2003). Evidence for diversifying selection on erythrocyte-binding antigens of Plasmodium falciparum and P. vivax. Genetics.

[107] Verra F., Chokejindachai W., Weedall G.D., Polley S.D., Mwangi T.W., Marsh K., Conway D.J. (2006). Contrasting signatures of selection on the Plasmodium falciparum erythrocyte binding antigen gene family. Mol. Biochem. Parasitol..

[108] Guy A.J., Irani V., Beeson J.G., Webb B., Sali A., Richards J.S., Ramsland P.A. (2018). Proteome-wide mapping of immune features onto Plasmodium protein three-dimensional structures. Sci. Rep..

[109] Kain K.C., Lanar D.E. (1991). Determination of genetic variation within Plasmodium falciparum by using enzymatically amplified DNA from filter paper disks impregnated with whole blood. J. Clin. Microbiol..

[110] Binks R.H., Baum J., Oduola A.M., Arnot D.E., Babiker H.A., Kremsner P.G., Roper C., Greenwood B.M., Conway D.J. (2001). Population genetic analysis of the Plasmodium falciparum erythrocyte binding antigen-175 (eba-175) gene. Mol. Biochem. Parasitol..

[111] Soulama I., Bigoga J.D., Ndiaye M., Bougouma E.C., Quagraine J., Casimiro P.N., Stedman T.T., Sirima S.B. (2011). Genetic diversity of polymorphic vaccine candidate antigens (apical membrane antigen-1, merozoite surface protein-3, and erythrocyte binding antigen-175) in Plasmodium falciparum isolates from western and central Africa. Am. J. Trop. Med. Hyg..

[112] Richards J.S., Stanisic D.I., Fowkes F.J., Tavul L., Dabod E., Thompson J.K., Kumar S., Chitnis C.E., Narum D.L., Michon P. (2010). Association between naturally acquired antibodies to erythrocyte-binding antigens of Plasmodium falciparum and protection from malaria and high-density parasitemia. Clin. Infect. Dis..

[113] Rodriguez L.E., Curtidor H., Urquiza M., Cifuentes G., Reyes C., Patarroyo M.E. (2008). Intimate molecular interactions of P. falciparum merozoite proteins involved in invasion of red blood cells and their implications for vaccine design. Chem. Rev..

[114] Patarroyo M.E., Arevalo-Pinzon G., Reyes C., Moreno-Vranich A., Patarroyo M.A. (2016). Malaria Parasite Survival Depends on Conserved Binding Peptides’ Critical Biological Functions. Curr. Issues Mol. Biol..

[115] Sakura T., Yahata K., Kaneko O. (2013). The upstream sequence segment of the C-terminal cysteine-rich domain is required for microneme trafficking of Plasmodium falciparum erythrocyte binding antigen 175. Parasitol. Int..

[116] Narum D.L., Haynes J.D., Fuhrmann S., Moch K., Liang H., Hoffman S.L., Sim B.K. (2000). Antibodies against the Plasmodium falciparum receptor binding domain of EBA-175 block invasion pathways that do not involve sialic acids. Infect. Immun..

[117] Jones T.R., Narum D.L., Gozalo A.S., Aguiar J., Fuhrmann S.R., Liang H., Haynes J.D., Moch J.K., Lucas C., Luu T. (2001). Protection of Aotus monkeys by Plasmodium falciparum EBA-175 region II DNA prime-protein boost immunization regimen. J. Infect. Dis..

[118] Sim B.K., Narum D.L., Chattopadhyay R., Ahumada A., Haynes J.D., Fuhrmann S.R., Wingard J.N., Liang H., Moch J.K., Hoffman S.L. (2011). Delineation of stage specific expression of Plasmodium falciparum EBA-175 by biologically functional region II monoclonal antibodies. PLoS ONE.

[119] Rodriguez L.E., Urquiza M., Ocampo M., Suarez J., Curtidor H., Guzman F., Vargas L.E., Trivinos M., Rosas M., Patarroyo M.E. (2000). Plasmodium falciparum EBA-175 kDa protein peptides which bind to human red blood cells. Parasitology.

