# Supplementary material for: Hotspots in Plasmodium and RBC Receptor-Ligand Interactions: Key Pieces for Inhibiting Malarial Parasite Invasion
Source: Int J Mol Sci. 2020 Jul 2;21(13):4729. doi: 10.3390/ijms21134729 (PMC7370042; doi:10.3390/ijms21134729)
Supplement: Supplementary file 1 [file ijms-21-04729-s001.pdf]

International Journal of *Molecular Sciences*

MDPI

Table S1. The main ligand-receptor interactions taking place during merozoite invasion of RBCs or reticulocytes.

Ligand	Receptor	Ligand Mrz location	Kd	Enzyme treatment			Invasion process involved	Interface interaction	Receptor-ligand structure	Ref
				T	C	N				
***Pf*MSP-1$_{83}$**	**GPA**	**Surface**	**ND**	**S**	**R**	**S**	IC	MSP1 N-terminus	ND	[1]
***Pf*MSP-1$_{42}$**	Heparin	Surface	ND	-	-	-	IC	ND	ND	[2]
***Pf*MSP-1$_{19}$**	Band3	Surface	ND	R	S	R	IC	MSP1$_{19}$ C-terminal binds to Band3 720–761aa	ND	[3]
***Pf*Rh1**	Y	Rhoptries	ND	R	R	S	AR	Rh1 RII-3 500-833aa	ND	[4,5]
***Pf*Rh2b**	Z	Rhoptries	ND	R	S	R	AR	ND	ND	[6,7]
***Pf*Rh4**	CR1	Rhoptries	2.9 ± 0.2 μM	S	S	R	AR	Rh4 ^{328}N–^{588}D aa binds to CR1^{18}D-^{20}F aa	ND	[8–12]
***Pf*Rh5**	BSG	Rhoptries	1.12 ± 0.09 μM	R	R	S / R	AR	Detailed in Figure 5	X-ray crystallography PDB: 4U0Q	[13]
***Pf*CyRPA**	Ternary complex with Rh5 and Ripr	Microneme	ND	-	-	-	AR	Cyrpa ^{393}L, ^{397}L, ^{494}F and ^{498}I aa form a Rh5 groove in contact with ^{185}Y, ^{187}F and ^{226}F presented by CyRPA loops β4 and β 4–β 5	Cryo-EM PDB: 6MPV	[14,15]
***Pf*Ripr**	Ternary complex with Rh5 and CyRPA	Microneme	ND	-	-	-	AR	Ripr 196-211 aa binds to CyRPA blade 6	Cryo-EM PDB: 6MPV	[14]
***Pf*EBA-175**	GPA	Microneme	~0.26 μM	S	R	S	AR	Detailed in Figure 6	X-ray crystallography PDB: 1ZRL and 1ZRO	[16]

***Pf*EBA-140**	GPC	Microneme	ND	S	R	S	AR	Both EBA-140 DBL domains	ND	[17,18]
***Pf*EBA-181/**	Sialic residues - Band 4.1	Microneme	745 nM	R	S	S	AR	EBA-181 binds to Band 4.1 (10 kDa domain 404-471aa)	ND	[19]
***Pf*EBL-1**	GPB	Microneme	ND	R	S	S	AR	EBL-1 region 2	ND	[20]
***Pf*AMA-1**	Kx	Microneme	ND	R	S	S	TJF	AMA1 domain III	ND	[21]
***Pf*TRAMP**	SEMA7A/ Aldolase	Microneme	1.18 ± 0.40 µM	-	-	-	AR and TJF	The two TSR domains ligand / entire SEMA7A ectodomain	ND	[22]
***Pv*MSP-1$_{19}$**	GPA-Band 3?	Surface	ND	S	R	S	IC	*Pv*MSP1$_{19}$ (C-terminal region) EGF-like motifs 1 and 2 bind to RBC	ND	[23,24]
***Pv*MSP-9**	Band3?	Surface	ND	R	S	R	IC	ND	ND	[25]
***Pv*TRAg36**	Band3	Surface	ND	R	S	R	IC	ND	ND	
***Pv*TRAg38**	Band3<br>BSG	Surface	Band 3 and the M-*Pv*TRAg38 fragment 7.39 ± 1.24 x 10^8M<br>BSG and the *Pv*TRAg 38 3.0 ± 0.68 10 ^{6} M	R<br>R	S<br>R	R<br>S	IC	*Pv*TRAg38 (^{197}KWVQWKNDKIRSW LSSEW^{214}) / Band 3<br>*Pv*TRAg38 (161TQWGNWIKTEGR KILEAQ178) / Basigin	ND	[26,27]
***Pv*AMA1**	GPB?	Surface	1.9 ± 0.34 µM	R	S	S	TJF	*Pv*AMA-DI-II (^{81}EVENAKYRIPAGRCP VFGKG^{100}) binding to young reticulocytes (CD71$^+$CD45$^-$)	ND	[28]
***Pv*RBP2b**	TfR	Rhoptries		S	S	R	AR	Ternary complex TfR1-Tf-*Pv*RBP2b TfR1TfR1 (residues 120–760) binding to two iron-	Cryo-EM PDB: 6D03, 6D04 and 6D05	[29,30]

								loaded Tf molecules (residues 1–679) with two *Pv*RBP2b molecules (residues 168–633) bound on either side.		
**DBP1**	DARC	Microneme	8.7 nM	R	S	S	TJF	Detailed in Figures 2 and 3	NMR, X-ray crystallography PDB: 3RRC, 4NUU, 4NUV, 5F3J, 6OAN, 6OAO 6R2S	[31–33]

ND: not determined**; Kd:** dissociation constant; **T**: trypsin; **C**: chymotrypsin; **N**: neuraminidase; **IC:** initial contact; **AR:** apical reorientation; **TJF:** tight-junction formation. GPC (Glycophorin C); TfR (Transferrin Receptor).

References

1. Baldwin, M.R.; Li, X.; Hanada, T.; Liu, S.C.; Chishti, A.H., Merozoite surface protein 1 recognition of host glycophorin A mediates malaria parasite invasion of red blood cells. *Blood* **2015,** *125*, 2704–2711.
2. Boyle, M.J.; Richards, J.S.; Gilson, P.R.; Chai, W.; Beeson, J.G., Interactions with heparin-like molecules during erythrocyte invasion by Plasmodium falciparum merozoites. *Blood* **2010,** *115*, 4559–4568.
3. Goel, V.K.; Li, X.; Chen, H.; Liu, S.C.; Chishti, A.H.; Oh, S.S., Band 3 is a host receptor binding merozoite surface protein 1 during the Plasmodium falciparum invasion of erythrocytes. *Proc. Natl Acad. Sc.i USA* **2003,** *100*, 5164–5169.
4. Gao, X.; Yeo, K.P.; Aw, S.S.; Kuss, C.; Iyer, J.K.; Genesan, S.; Rajamanonmani, R.; Lescar, J.; Bozdech, Z.; Preiser, P.R., Antibodies targeting the PfRH1 binding domain inhibit invasion of Plasmodium falciparum merozoites. *PLoS Pathog.* **2008,** *4*, doi: 10.1371/journal.ppat.1000104.
5. Triglia, T.; Duraisingh, M.T.; Good, R.T.; Cowman, A.F., Reticulocyte-binding protein homologue 1 is required for sialic acid-dependent invasion into human erythrocytes by Plasmodium falciparum. *Mol. Microbiol.* **2005,** *55*, 162–174.
6. Sahar, T.; Reddy, K.S.; Bharadwaj, M.; Pandey, A.K.; Singh, S.; Chitnis, C.E.; Gaur, D., Plasmodium falciparum reticulocyte binding-like homologue protein 2 (PfRH2) is a key adhesive molecule involved in erythrocyte invasion. *PLoS ONE* **2011,** *6*, 10.1371/journal.pone.0017102.
7. Duraisingh, M.T.; Triglia, T.; Ralph, S.A.; Rayner, J.C.; Barnwell, J.W.; McFadden, G.I.; Cowman, A.F., Phenotypic variation of Plasmodium falciparum merozoite proteins directs receptor targeting for invasion of human erythrocytes. *EMBO J.* **2003,** *22*, 1047–1057.
8. Cockburn, I.A.; Mackinnon, M.J.; O'Donnell, A.; Allen, S.J.; Moulds, J.M.; Baisor, M.; Bockarie, M.; Reeder, J.C.; Rowe, J.A., A human complement receptor 1 polymorphism that reduces Plasmodium falciparum rosetting confers protection against severe malaria. *Proc. Natl Acad. Sci. USA* **2004,** *101*, 272–277.
9. Tham, W.H.; Wilson, D.W.; Lopaticki, S.; Schmidt, C.Q.; Tetteh-Quarcoo, P.B.; Barlow, P.N.; Richard, D.; Corbin, J.E.; Beeson, J.G.; Cowman, A.F., Complement receptor 1 is the host erythrocyte receptor for Plasmodium falciparum PfRh4 invasion ligand. *Proc. Natl Acad. Sci. USA* **2010,** *107*, 17327–17332.
10. Park, H.J.; Guariento, M.; Maciejewski, M.; Hauhart, R.; Tham, W.H.; Cowman, A.F.; Schmidt, C.Q.; Mertens, H.D.; Liszewski, M.K.; Hourcade, D.E.; et al. Using mutagenesis and structural biology to map the binding site for the Plasmodium falciparum merozoite protein PfRh4 on the human immune adherence receptor. *J. Biol. Chem.* **2014,** *289*, 450–463.
11. Gaur, D.; Singh, S.; Singh, S.; Jiang, L.; Diouf, A.; Miller, L.H., Recombinant Plasmodium falciparum reticulocyte homology protein 4 binds to erythrocytes and blocks invasion. *Proc. Natl Acad. Sci. USA* **2007,** *104*, 17789–17794.
12. Spadafora, C.; Awandare, G.A.; Kopydlowski, K.M.; Czege, J.; Moch, J.K.; Finberg, R.W.; Tsokos, G.C.; Stoute, J.A., Complement receptor 1 is a sialic acid-independent erythrocyte receptor of Plasmodium falciparum. *PLoS Pathog.* **2010,** *6*, doi: 10.1371/journal.ppat.1000968.

13. Chen, L.; Xu, Y.; Healer, J.; Thompson, J.K.; Smith, B.J.; Lawrence, M.C.; Cowman, A.F., Crystal structure of PfRh5, an essential P. falciparum ligand for invasion of human erythrocytes. *Elife* **2014,** *3*, doi: 10.7554/eLife.04187.
14. Wong, W.; Huang, R.; Menant, S.; Hong, C.; Sandow, J.J.; Birkinshaw, R.W.; Healer, J.; Hodder, A.N.; Kanjee, U.; Tonkin, C.J.; et al. Structure of Plasmodium falciparum Rh5-CyRPA-Ripr invasion complex. *Nature* **2019,** *565*, 118–121.
15. Volz, J.C.; Yap, A.; Sisquella, X.; Thompson, J.K.; Lim, N.T.; Whitehead, L.W.; Chen, L.; Lampe, M.; Tham, W.H.; Wilson, D.; et al. Essential Role of the PfRh5/PfRipr/CyRPA Complex during Plasmodium falciparum Invasion of Erythrocytes. *Cell Host Microbe.* **2016,** *20*, 60–71.
16. Tolia, N.H.; Enemark, E.J.; Sim, B.K.; Joshua-Tor, L., Structural basis for the EBA-175 erythrocyte invasion pathway of the malaria parasite Plasmodium falciparum. *Cell* **2005,** *122*, 183–193.
17. Lin, D.H.; Malpede, B.M.; Batchelor, J.D.; Tolia, N.H., Crystal and solution structures of Plasmodium falciparum erythrocyte-binding antigen 140 reveal determinants of receptor specificity during erythrocyte invasion. *J. Biol. Chem.* **2012,** *287*, 36830–36836.
18. Malpede, B.M.; Lin, D.H.; Tolia, N.H., Molecular basis for sialic acid-dependent receptor recognition by the Plasmodium falciparum invasion protein erythrocyte-binding antigen-140/BAEBL. *J. Biol. Chem.* **2013,** *288*, 12406–12415.
19. Lanzillotti, R.; Coetzer, T.L., The 10 kDa domain of human erythrocyte protein 4.1 binds the Plasmodium falciparum EBA-181 protein. *Malar J.* **2006**, *5*, doi: 10.1186/1475-2875-5-100
20. Mayer, D.C.; Cofie, J.; Jiang, L.; Hartl, D.L.; Tracy, E.; Kabat, J.; Mendoza, L.H.; Miller, L.H., Glycophorin B is the erythrocyte receptor of Plasmodium falciparum erythrocyte-binding ligand, EBL-1. *Proc. Natl Acad. Sci. USA* **2009,** *106*, 5348–5352.
21. Kato, K.; Mayer, D.C.; Singh, S.; Reid, M.; Miller, L.H., Domain III of Plasmodium falciparum apical membrane antigen 1 binds to the erythrocyte membrane protein Kx. *Proc. Natl Acad. Sci. USA* **2005,** *102*, 5552–5557.
22. Bartholdson, S.J.; Bustamante, L.Y.; Crosnier, C.; Johnson, S.; Lea, S.; Rayner, J.C.; Wright, G.J., Semaphorin-7A is an erythrocyte receptor for P. falciparum merozoite-specific TRAP homolog, MTRAP. *PLoS Pathog.* **2012,** *8*, doi: 10.1371/journal.ppat.1003031.
23. Han, H.J.; Park, S.G.; Kim, S.H.; Hwang, S.Y.; Han, J.; Traicoff, J.; Kho, W.G.; Chung, J.Y., Epidermal growth factor-like motifs 1 and 2 of Plasmodium vivax merozoite surface protein 1 are critical domains in erythrocyte invasion. *Biochem. Biophys. Res. Commun* **2004,** *320*, 563–570.
24. Rodriguez, L.E.; Urquiza, M.; Ocampo, M.; Curtidor, H.; Suarez, J.; Garcia, J.; Vera, R.; Puentes, A.; Lopez, R.; Pinto, M.; et al. Plasmodium vivax MSP-1 peptides have high specific binding activity to human reticulocytes. *Vaccine* **2002,** *20*, 1331–1339.
25. Alam, M.S.; Rathore, S.; Tyagi, R.K.; Sharma, Y.D., Host-parasite interaction: Multiple sites in the Plasmodium vivax tryptophan-rich antigen PvTRAg38 interact with the erythrocyte receptor band 3. *FEBS Letters* **2016,** *590*, 232–241.
26. Alam, M.S.; Choudhary, V.; Zeeshan, M.; Tyagi, R.K.; Rathore, S.; Sharma, Y.D., Interaction of Plasmodium vivax Tryptophan-rich Antigen PvTRAg38 with Band 3 on Human Erythrocyte Surface Facilitates Parasite Growth. *J. Biol. Chem.* **2015,** *290*, 20257–20272.
27. Rathore, S.; Dass, S.; Kandari, D.; Kaur, I.; Gupta, M.; Sharma, Y.D., Basigin Interacts with Plasmodium vivax Tryptophan-rich Antigen PvTRAg38 as a Second Erythrocyte Receptor to Promote Parasite Growth. *J. Biol. Chem.* **2017,** *292*, 462-476.

28. Arevalo-Pinzon, G.; Bermudez, M.; Hernandez, D.; Curtidor, H.; Patarroyo, M.A., Plasmodium vivax ligand-receptor interaction: PvAMA-1 domain I contains the minimal regions for specific interaction with CD71+ reticulocytes. *Sci. Rep.* **2017,** *7*, doi: 10.1038/s41598-017-10025-6.
29. Gruszczyk, J.; Kanjee, U.; Chan, L.J.; Menant, S.; Malleret, B.; Lim, N.T.Y.; Schmidt, C.Q.; Mok, Y.F.; Lin, K.M.; Pearson, R.D.; et al. Transferrin receptor 1 is a reticulocyte-specific receptor for Plasmodium vivax. *Science* **2018,** *359*, 48–55.
30. Gruszczyk, J.; Huang, R.K.; Chan, L.J.; Menant, S.; Hong, C.; Murphy, J.M.; Mok, Y.F.; Griffin, M.D.W.; Pearson, R.D.; Wong, W.; et al. Cryo-EM structure of an essential Plasmodium vivax invasion complex. *Nature* **2018,** *559*, 135-139.
31. Batchelor, J.D.; Malpede, B.M.; Omattage, N.S.; DeKoster, G.T.; Henzler-Wildman, K.A.; Tolia, N.H., Red blood cell invasion by Plasmodium vivax: Structural basis for DBP engagement of DARC. *PLoS Pathog.* **2014,** *10*, doi: 10.1371/journal.ppat.1003869
32. Batchelor, J.D.; Zahm, J.A.; Tolia, N.H., Dimerization of Plasmodium vivax DBP is induced upon receptor binding and drives recognition of DARC. *Nat. Struct. Mol. Biol.* **2011,** *18*, 908–914.
33. Hans, D.; Pattnaik, P.; Bhattacharyya, A.; Shakri, A.R.; Yazdani, S.S.; Sharma, M.; Choe, H.; Farzan, M.; Chitnis, C.E., Mapping binding residues in the Plasmodium vivax domain that binds Duffy antigen during red cell invasion. *Mol. Microbiol.* **2005,** *55*, 1423–1434.

© 2020 by the authors. Submitted for possible open access publication under the terms and conditions of the Creative Commons Attribution (CC BY) license (http://creativecommons.org/licenses/by/4.0/).
